# Comparison of NF-κB from the protists *Capsaspora owczarzaki* and *Acanthoeca spectabilis* reveals extensive evolutionary diversification of this transcription factor

**DOI:** 10.1038/s42003-021-02924-2

**Published:** 2021-12-16

**Authors:** Leah M. Williams, Sainetra Sridhar, Jason Samaroo, Jada Peart, Ebubechi K. Adindu, Anvitha Addanki, Christopher J. DiRusso, BB522 Molecular Biology Laboratory, Dana H. M. Alburi, Ludmila Anisimov, Aria Y. Armstrong, Sydney J. Badger, Elham Banaie, Joana A. Barbosa Teixeira, Madeleine T. Billingsley, Anoush Calikyan, Yinxing Chen, Aidan B. Coia, Daniel Cutillo, Breanna R. Dooling, Parth P. Doshi, Kyra R. Dubinsky, Berta Escude Velasco, Jabari R. Evans, Jasmine Gordon, Huibo Guan, Spiro N. Haliotis, Niccolas T. Hood, Yen-Chun Huang, Wenjing Jiang, Isabelle C. Kreber, Ekin B. Kurak, Cheng-Che Lee, Tanner M. Lehmann, Savina J. W. Lin, Edward Liu, Kevin Liu, Yen-Yu Liu, Alexandra L. Luther, Alexa A. Macgranaky-Quaye, Daniel J. Magat, Lauren E. Malsick, Parmida Masoudi, Parsida Masoudi, Chad R. H. Max, Ethan Z. McCaslin, Eleanor T. McGeary, Kathleen M. McLaughlin, Victoria S. A. Momyer, Lake D. Murphy, Sonny V. Nguyen, Kareemah Ni, Leon Novak, Roberto Nunes Campos E. Santos, Yemi D. Osayame, Jun Bai Park Chang, Harshal M. Patel, Tony V. Pham, Sheila M. Phillips, Jhonathan Perea Piedrahita, Tricia L. Post, Rebecca A. Prather, Pauline I. Reck, Jaime A. Rodriguez, Kirquenique A. Rolle, Joseph A. Salzo, Kathryn M. Satko, Davis G. Settipane, Kara J. Sevola, Mithil V. Shah, Viktoriya Skidanova, Georgia M. Snyder, Rebecca J. Sprague, Ryan A. Stagg, Danielle Tong, Andreas A. Towers, Nicholas W. Turgiss, Natalie S. Wheeler, Ann S. Yung, Pablo J. Aguirre Carrión, Nahomie Rodriguez-Sastre, Trevor Siggers, Thomas D. Gilmore

**Affiliations:** 1 Department of Biology, Boston University, Boston, MA USA; 2 Program in Biochemistry & Molecular Biology, Boston University, Boston, MA USA

**Keywords:** Cell signalling, NF-kappaB

## Abstract

We provide a functional characterization of transcription factor NF-κB in protists and provide information about the evolution and diversification of this biologically important protein. We characterized NF-κB in two protists using phylogenetic, cellular, and biochemical techniques. NF-κB of the holozoan *Capsaspora owczarzaki* (*Co*) has an N-terminal DNA-binding domain and a C-terminal Ankyrin repeat (ANK) domain, and its DNA-binding specificity is more similar to metazoan NF-κB proteins than to Rel proteins. Removal of the ANK domain allows *Co*-NF-κB to enter the nucleus, bind DNA, and activate transcription. However, C-terminal processing of *Co*-NF-κB is not induced by IκB kinases in human cells. Overexpressed *Co*-NF-κB localizes to the cytoplasm in *Co* cells. *Co*-NF-κB mRNA and DNA-binding levels differ across three *Capsaspora* life stages. RNA-sequencing and GO analyses identify possible gene targets of *Co*-NF-κB. Three NF-κB-like proteins from the choanoflagellate *Acanthoeca spectabilis* (*As*) contain conserved Rel Homology domain sequences, but lack C-terminal ANK repeats. All three *As*-NF-κB proteins constitutively enter the nucleus of cells, but differ in their DNA-binding abilities, transcriptional activation activities, and dimerization properties. These results provide a basis for understanding the evolutionary origins of this key transcription factor and could have implications for the origins of regulated immunity in higher taxa.

## Abstract (web summary)

Transcription factor NF-ĸB is a key regulator of immunity in mammals, but its function in protists like *Capsaspora* and choanoflagellates is not known. Here, Leah Williams et al. characterize and compare the structure, activity, and regulation of NF-ĸB from *Capsaspora* and one choanoflagellate, providing further insight into the origins of NF-ĸB.

## Introduction

Transcription factor NF-κB (Nuclear Factor-κB) has been extensively studied for its roles in development and immunity in animals from sponges to humans^1–3^. More recently, it has been discovered that certain single-celled eukaryotes, namely *Capsaspora owczarzaki* and some choanoflagellates, also contain genes encoding NF-κB-like proteins^4–6^. To further our understanding of the origins and diversity of NF-κB, we have carried out a functional characterization of this important transcription factor in single-celled protists.

Protists comprise a large group of diverse eukaryotes that are either unicellular or multicellular with poorly differentiated tissue^7^. Two protists that have been studied reasonably well are the monotypic genus *Capsaspora owczarzaki* and the taxonomic class of choanoflagellates, both of which are in the monophyletic Filozoa clade within the Opisthokonta^8^.

*Capsaspora* is a close relative of animals (i.e., basal to sponges) and is classified in the Filasterea, which is an independent group within the clade Filozoa that is sister to choanoflagellates and metazoans^9–13^. The genome of *Capsaspora* encodes many proteins involved in metazoan multicellular processes such as integrins, protein tyrosine kinases, and transcription factors, including NF-κB^6^. *Capsaspora* was originally discovered as an ameba-like symbiont in the hemolymph of the fresh-water snail *Biomphalaria glabrata*^9,14,15^. The life cycle of *Capsaspora* has been shown to have three different cell configurations^16^. Under in vitro culture conditions, *Capsaspora* grow primarily as filopodial cells, which attach to the substrate and undergo active replication until the end of the exponential growth phase. Then, cells start to detach, retracting their branching filopodia, and forming cysts. During this cystic phase, cell division stops. Alternatively, filopodia cells can form a multicellular aggregative structure by secreting an unstructured extracellular matrix that promotes aggregation but prevents direct cell-cell contact^16^. Furthermore, each of the three life stages of *Capsaspora* has been reported to have distinct transcriptomic^16^ and proteomic/phosphoproteomic profiles^17^. Little is known about the molecular details of how these life-stage processes and transitions are regulated.

A second group of protists that are also widely regarded as close relatives of animals are the choanoflagellates, which comprise over 125 species of free-living unicellular and colonial organisms distributed in nearly every aquatic environment^18^. The first two published choanoflagellate genomes (of *Monosiga brevicollis* and *Salpingoeca rosetta*) did not contain NF-κB homologs^19,20^. However, Richter et al.^5^ subsequently reported the transcriptomes of 19 additional choanoflagellates, and 12 of these species expressed NF-κB-like genes, including several with multiple NF-κB-like transcripts.

Proteins in the NF-κB superfamily have a conserved N-terminal Rel Homology Domain (RHD), which contains sequences required for dimerization, DNA binding, and nuclear localization^1^. All NF-κB proteins bind to a collection of related DNA sites known as κB sites, and NF-κB proteins bind DNA as either homodimers or heterodimers. In animals from insects to humans, there are two subclasses of NF-κB proteins that differ in amino acid sequence relatedness, C-terminal domain sequences, and DNA-binding site preferences^1^. One subclass includes the traditional NF-κB proteins (p52/p100, p50/p105, Relish) that contain C-terminal inhibitory sequences known as Ankyrin (ANK) repeats, whereas the second class consists of the Rel proteins (RelA, RelB, cRel, Dorsal, Dif) that contain C-terminal transactivation domains. Among early-branching organisms—including cnidarians, poriferans, and some protists—only traditional NF-κB-like proteins have been found^3^.

In most multicellular animals, the activity of NF-κB proteins is regulated by subcellular localization, wherein an inactive NF-κB dimer is sequestered in the cytoplasm due to interaction with inhibitory IκB sequences (including the C-terminal ANK repeats of NF-κB proteins). Many upstream signals, including the binding of various ligands to conserved receptors (e.g., Toll-like Receptors (TLRs), Interleukin-1 receptors (IL-1Rs), and tumor necrosis factor receptors (TNFRs)), initiate a signal transduction pathway culminating in activation and nuclear translocation of NF-κB^1,2^. In the non-canonical pathway, the translocation of NF-κB from the cytoplasm to the nucleus is commenced by the phosphorylation of serine residues C-terminal to the ANK repeats, which leads to removal of the C-terminal ANK repeats by a proteasomal processing that begins at the C terminus and stops within a glycine-rich region (GRR) near the end of the RHD^21^. Removal of the ANK repeat sequences allows NF-κB to translocate to the nucleus, bind DNA, and activate the transcription of target genes for a given biological outcome.

Herein, we have characterized molecular functions of transcription factor NF-κB from two unicellular protists using phylogenetic, cellular, and biochemical techniques. We find that the *Capsaspora*NF-κB protein (*Co*-NF-κB) requires removal of C-terminal ANK repeats to enter the nucleus, bind DNA, and activate transcription. Furthermore, we show that the multiple NF-κBs of a single choanoflagellate (*Acanthoeca spectabilis)* can form heterodimers, suggesting that some choanoflagellates contain subclasses of interacting NF-κBs, as are found in vertebrates and flies. Overall, these results provide a functional characterization of NF-κB in a taxon other than Animalia.

## Results

### Protist NF-κB proteins vary in domain structure and choanoflagellates show evidence of gene duplication

**Fig. 1:**
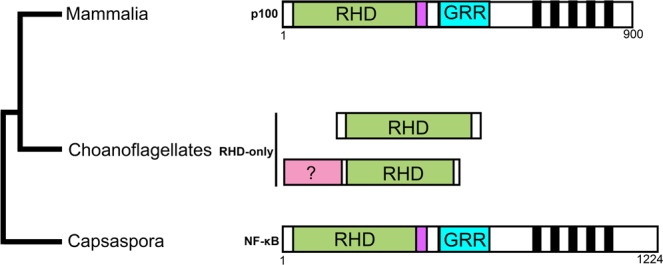
Protist and mammalian NF-κB proteins differ in domain structures. Shown are the general domain structures of *Capsaspora* NF-κB and the choanoflagellate RHD-only proteins as compared to mammalian NF-κBs. Green, RHD (Rel Homology Domain); Purple, nuclear localization sequence; Blue, GRR (glycine-rich region); Black bars, Ankyrin repeats; Pink, sequences in choanoflagellates that are not typically seen in other organisms.

Sebé-Pedrós et al.^6^ reported the presence of a single gene encoding an NF-κB-like protein in *C. owczarzaki* (*Co*). Like most other known basal NF-κB proteins, *Co*-NF-κB has an N-terminal RHD, followed by a glycine-rich region (GRR), and five C-terminal ANK repeats^6,22–26^. Despite the similarity in domain structure, the amino acid sequence of *Co-*NF-κB is only ~30% similar to mammalian NF-κBs (i.e., the p100/p105 NF-κB proteins), and this similarity is primarily due to sequence conservation of the ANK repeats. Indeed, less than 50 of ~300 amino acids in the RHD are >80% conserved among human and protist NF-κBs (Supplementary Fig. 1a). *Co*-NF-κB is also larger than other NF-κB homologs, primarily due to additional residues C-terminal to the ANK repeats (Fig. 1).

Of the 12 choanoflagellate species that are known to contain NF-κB-like genes, there are between one and three NF-κB genes present^5,19,20^. A phylogenetic comparison suggests that some of these choanoflagellate NF-κBs arose by gene duplications within a given species because the multiple NF-κBs from a given species cluster closely to each other (Supplementary Fig. 1b and ref. ^3^). Nevertheless, other choanoflagellates have multiple NF-κBs that cluster separately with individual NF-κBs of other choanoflagellates^3^, suggesting that different classes of NF-κBs exist in certain choanoflagellates.

Unlike most early-branching metazoans, choanoflagellates NF-κB transcripts primarily encode RHD sequences, with no C-terminal GRRs or ANK repeats. However, some choanoflagellate NF-κBs contain extended N termini with homology to sequences that are not normally associated with NF-κBs in vertebrates (Fig. 1, pink bar).

### DNA binding, nuclear translocation, and transactivation by *Co*-NF-κB

**Fig. 2:**
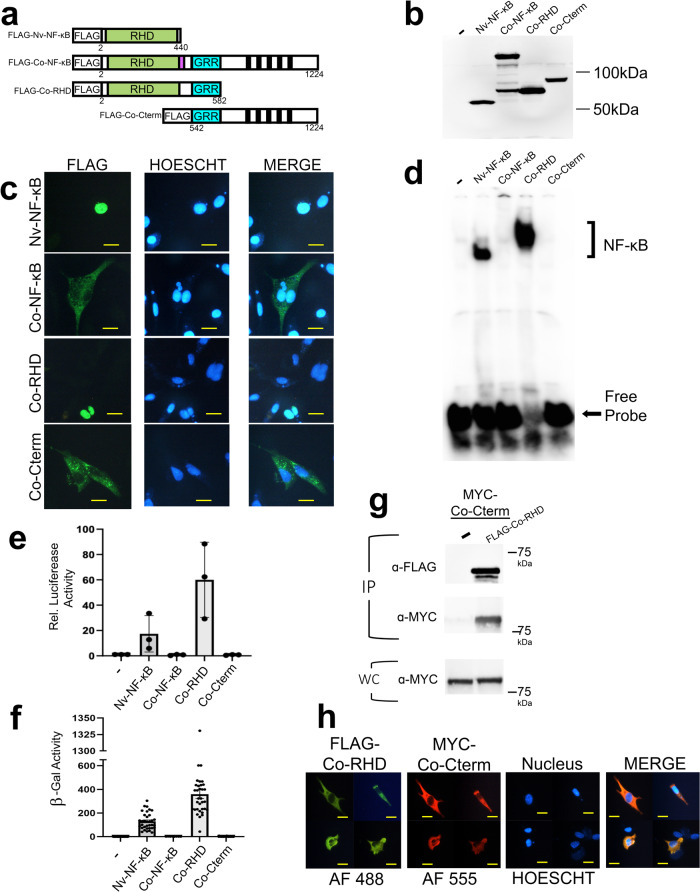
DNA-binding, subcellular localization, and transcriptional activation activities of *Co*-NF-κB. **a** FLAG-tagged expression vectors used in these experiments. From top to bottom, the drawings depict the naturally shortened *Nv*-NF-κB, the full-length *Co*-NF-κB protein, an N-terminal-only mutant containing the RHD and GRR (*Co*-RHD), and a C-terminal-only mutant containing the ANK repeats and other C-terminal sequences (*Co*-Cterm). **b** Anti-FLAG Western blot of lysates of HEK 293T cells transfected with the indicated expression vectors or the vector control (-). Raw image is shown in Supplementary Fig. 10. **c** Indirect immunofluorescence of DF-1 chicken fibroblasts transfected with the indicated expression vectors. Cells were then stained with anti-FLAG antiserum (left panels) and Hoechst (middle panels), and then merged in the right panels. Yellow scale bar is 10 µm. **d** A κB-site electromobility shift assay (EMSA) using a palindromic κB-site probe (GGGAATTCCC) and each of the indicated lysates from **b**. The NF-κB complexes and free probe are indicated by arrows. Raw image is shown in Supplementary Fig. 10. **e** A κB-site luciferase reporter gene assay was performed with the indicated proteins in HEK 293 cells. Luciferase activity is relative (Rel.) to that seen with the empty vector control (-) (set at 1.0). Values are averages of n = 3 biological replicates per sample, each of which was performed in triplicate, and are reported with standard error. Raw data are in Supplementary Data 5. **f** A GAL4-site *LacZ* reporter gene assay was performed in yeast Y190 cells with the indicated GAL4-fusion proteins or the GAL4 vector alone control (-). Values are the average β-gal units for n = 34 samples, except for 8 samples for *Co*-NF-κB, and are presented with standard errors. Raw data are in Supplementary Data 5. **g** Co-immunoprecipitation (IP) assays of MYC-tagged *Co*-Cterm. MYC-*Co*-Cterm was co-transfected in HEK 293T cells with pcDNA FLAG or FLAG-*Co*-RHD as indicated. An IP using anti-FLAG beads was next performed. After isolating proteins on FLAG beads and separating proteins on SDS-polyacrylamide gels, Western blotting with anti-FLAG (top) and anti-MYC (middle) antibodies was then performed. An anti-MYC Western blot of the whole-cell (WC) lysates was also performed (bottom). Raw image is shown in Supplementary Fig. 10. **h** Indirect immunofluorescence of DF-1 chicken fibroblasts transfected with the indicated expression vectors. Shown are four representative cells that were co-stained with anti-FLAG antiserum (left panel, Alexa fluor-488), anti-MYC antiserum (second panel, Alexa flour-555), Hoechst (third panel), and then merged (right panel).

To investigate properties of *Co-*NF-κB in cells, we created pcDNA-FLAG vectors for full-length*Co*-NF-κB and two truncation mutants, one (FLAG-*Co*-RHD) containing the N-terminal RHD sequences including the NLS and the GRR, and a second (FLAG-*Co*-Cterm) consisting of the C-terminal ANK repeat sequences and downstream residues (Fig. 2a). As a control, we used the active, naturally truncated sea anemone *Nematostella vectensis* (*Nv*) FLAG-tagged*Nv*-NF-κB protein that we have characterized previously^26^ (Fig. 2a). As shown by anti-FLAG Western blotting, each plasmid expressed a protein of the appropriate size when transfected into HEK 293T cells (Fig. 2b, Supplementary Table 1).

For some sponge and cnidarian NF-κBs, removal of C-terminal ANK repeat sequences is required for nuclear localization when expressed in vertebrate cells^22–24^. Therefore, we transfected each of our FLAG expression plasmids into DF-1 chicken fibroblast cells and performed indirect immunofluorescence using anti-FLAG antiserum (Fig. 2c). Full-length*Co*-NF-κB and *Co*-Cterm were both located primarily in the cytoplasm of >94% of cells (Supplementary Table 2). In contrast, the *Co*-RHD and control *Nv*-NF-κB proteins were both primarily nuclear, as evidenced by co-localization with the Hoechst-stained nuclei. Thus, the removal of the ANK repeats allows *Co*-NF-κB to enter the nucleus, consistent with what is seen with other metazoan RHD-ANK bipartite NF-κB proteins.

To assess the DNA-binding activity of *Co*-NF-κB proteins, whole-cell extracts from 293T cells transfected with each FLAG construct were analyzed in an electrophoretic mobility shift assay (EMSA) using a consensus κB-site probe. Extracts containing overexpressed *Nv*-NF-κB and *Co*-RHD showed a prominent bound κB-site complex, whereas extracts containing full-length*Co*-NF-κB and *Co*-Cterm showed essentially no κB site-binding activity (Fig. 2d).

We also assessed the ability of *Co*-NF-κB proteins to activate transcription in HEK 293 cells using a κB-site luciferase reporter plasmid (Fig. 2e). *Co*-RHD and *Nv*-NF-κB activated transcription well above empty vector control levels (i.e., *Co*-RHD was ~60-fold above the vector alone control). In contrast, full-length and Cterm *Co*-NF-κB proteins showed little to no ability to activate transcription. Thus, the ability to activate transcription of a κB-site gene locus appears to be a property of sequences within the N-terminal half of *Co*-NF-κB.

To assess the inherent ability of *Co*-NF-κB proteins to activate transcription, we measured their abilities to activate transcription in a *lacZ* reporter gene assay in yeast using a GAL4-site reporter (Fig. 2f). When fused to the DNA-binding domain of GAL4, the *Co*-RHD protein activated transcription strongly in yeast, nearly 400-fold above the GAL4 (aa 1-147) alone negative control. The potent transactivating ability of the GAL4-*Co*-RHD fusion in yeast suggests that transactivation is an intrinsic property of the RHD sequences. The *Co*-Cterm sequences did not activate transcription in yeast, and the full-length*Co*-NF-κB protein had greatly reduced transactivation ability compared to the *Co*-RHD domain sequences in yeast (Fig. 2f), indicating that C-terminal sequences can directly block transactivation by the *Co*-RHD sequences.

To investigate the mechanism by which the C-terminal sequences of *Co*-NF-κB inhibit the RHD sequences, we assessed interactions between C-terminal and RHD sequences in two ways. First, a MYC-tagged*Co*-Cterm mutant was co-transfected with the FLAG-*Co*-RHD mutant into 293T cells. FLAG proteins were later immunoprecipitated using anti-FLAG beads, and the resulting immunoprecipitates were subjected to anti-MYC Western blotting (Fig. 2g). MYC-*Co*-Cterm was co-immunoprecipitated with FLAG-*Co*-RHD. The MYC-*Co*-Cterm was not seen when co-immunoprecipitations were performed with the empty vector control. Secondly, when MYC-*Co*-Cterm and FLAG-*Co*-RHD were co-expressed in DF-1 cells, both proteins were localized in the cytoplasm (Fig. 2h). In contrast, when expressed alone, FLAG-*Co*-RHD localized to the nucleus and MYC-*Co-*Cterm was cytoplasmic (Supplementary Fig. 2), indicating that the cytoplasmic *Co*-Cterm protein can block nuclear translocation of the *Co*-RHD.

The results in this section show that *Co*-RHD can bind DNA, activate transcription, and localize primarily to the nucleus. However, these activities of *Co*-RHD are inhibited by a direct interaction with C-terminal ANK repeat-containing sequences, which is consistent with findings with most NF-κBs from sponges to humans^22–24,26^.

### IKK-mediated processing of NF-κB appears to have evolved with the rise of multicellularity

Like *Co*-NF-κB, vertebrate NF-κB p100 requires the removal of its C-terminal ANK repeats to enter the nucleus^21^. Proteasome-mediated processing of p100 is initiated by phosphorylation of a C-terminal cluster of serine residues by an IκB kinase (IKK)^21^.

**Fig. 3:**
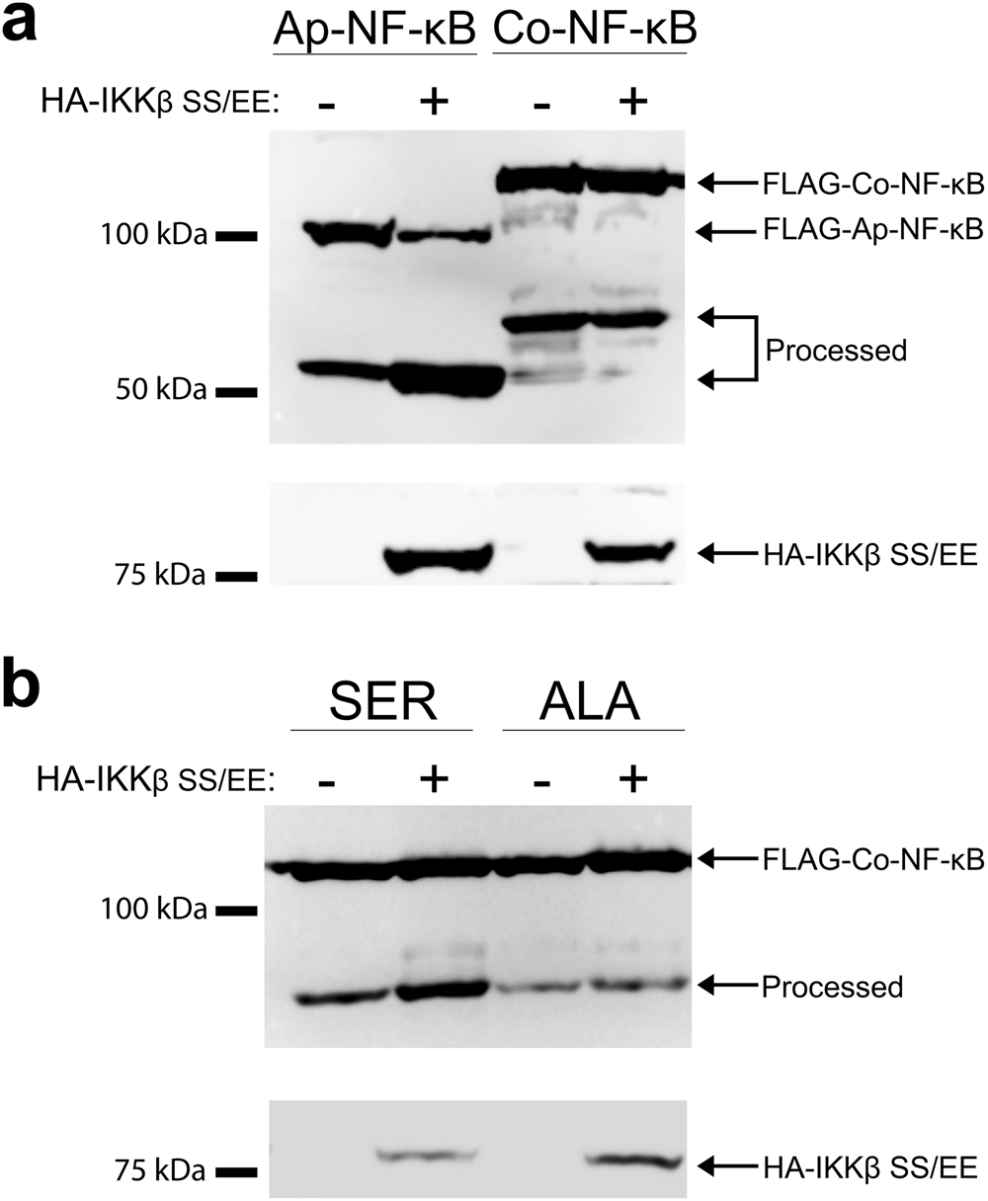
IKK does not induce processing of *Co*-NF-κB. **a** Co-transfection with IKKβ does not induce processing of FLAG-*Co*-NF-κB above what is seen with the vector alone control (-) in HEK 293T cells. Arrows indicate the HA-IKKβ (SS/EE) used in these assays (probed with anti-HA antiserum). Full-length FLAG-*Ap*-Co-NF-κB and FLAG-*Co*-NF-κB and their processed forms are also indicated (probed with anti-FLAG antiserum). Raw image is shown in Supplementary Fig. 11. **b** *Co*-NF-κB with C-terminal IKK target serines from *Aiptasia* NF-κB (*Co*-NF-κB-SER) or serine-to-alanine mutants (*Co*-NF-κB-ALA) were co-transfected into 293T cells with constitutively active human HA-IKKβ (SS/EE) or the vector alone control (-). Transfection of *Co*-NF-κB-SER and HA-IKKβ (SS/EE) resulted in the appearance of an increased amount of the lower band, but the alanine mutations (*Co*-NF-κB-ALA) did not show HA-IKKβ (SS/EE)-enhanced processing. Proteins were detected by anti-FLAG and anti-HA Western blotting as in **a**. Raw image is shown in Supplementary Fig. 11.

We have previously shown that some early-branching organisms, including NF-κB proteins from two cnidarians and one sponge, contain homologous C-terminal serines that can be phosphorylated by several IKKs to initiate proteasome-mediated processing in human cell culture assays^22–24^. Examination of the C-terminal sequences of *Co*-NF-κB failed to identify any C-terminal serine clusters similar to NF-κBs that undergo IKK-initiated processing. Nevertheless, we sought to experimentally assess whether IKK could induce processing of full-length*Co*-NF-κB. For these experiments, we co-transfected HEK 293T cells with *Co*-NF-κB and a constitutively active human IKKβ protein (IKKβ SS/EE) (Fig. 3a), as well as other IKKs including two from humans and one from a sea anemone (Supplementary Fig. 3a)^22–24^. As a positive control, the RHD-ANK repeat NF-κB protein from *Aiptasia* (*Ap*) was used, which has been shown to undergo IKK-induced processing in HEK 293T cells^24^. Although IKK induced processing of *Ap*-NF-κB, none of the IKKs induced processing of *Co*-NF-κB (Fig. 3a and Supplementary Fig. 3a), beyond the small amount of constitutive processing of *Co*-NF-κB that occurs even in the absence of IKK (Figs. 2b and 3a). Of note, the lower *Co*-NF-κB band seen in these Western blots was roughly the same size as the predicted RHD (Fig. 2b), and incubation of transfected cells with the proteasome inhibitor MG132 reduced the appearance of the lower band, suggesting that the lower band arises by proteasomal processing of full-length*Co*-NF-κB in 293T cells (Supplementary Fig. 3b).

To determine whether *Co*-NF-κB *could be* processed by an IKK-dependent mechanism, we created a mutant in which we replaced C-terminal sequences *Co*-NF-κB (downstream of the ANK repeats) with C-terminal sequences of the sea anemone *Aiptasia* (*Ap*)-NF-κB that contain conserved serines which can facilitate IKK-induced processing of *Ap*-NF-κB^24^. We termed this mutant *Co*-NF-κB-SER, and also created the analogous protein (*Co*-NF-κB-ALA) in which the relevant serines were replaced by non-phosphorylatable alanines. Co-expression of *Co*-NF-κB-SER with constitutively active human IKKβ (IKKβ SS/EE) resulted in increased amounts of the lower band, which was not seen with *Co*-NF-κB-ALA (Fig. 3b). Thus, the *Co*-NF-κB protein (consisting of the RHD, GRR, and ANK repeats) can undergo IKK-induced processing if supplied with a C terminus containing IKK target serine residues. However, the native *Co*-NF-κB protein does not appear to be susceptible to IKK-induced processing, which is consistent with the lack of any sequences encoding an IKK-like protein in the genome of *Capsaspora*.

### Exogenously expressed full-length and truncated versions of *Co*-NF-κB localize primarily to the cytoplasm in *Capsaspora* cells

**Fig. 4:**
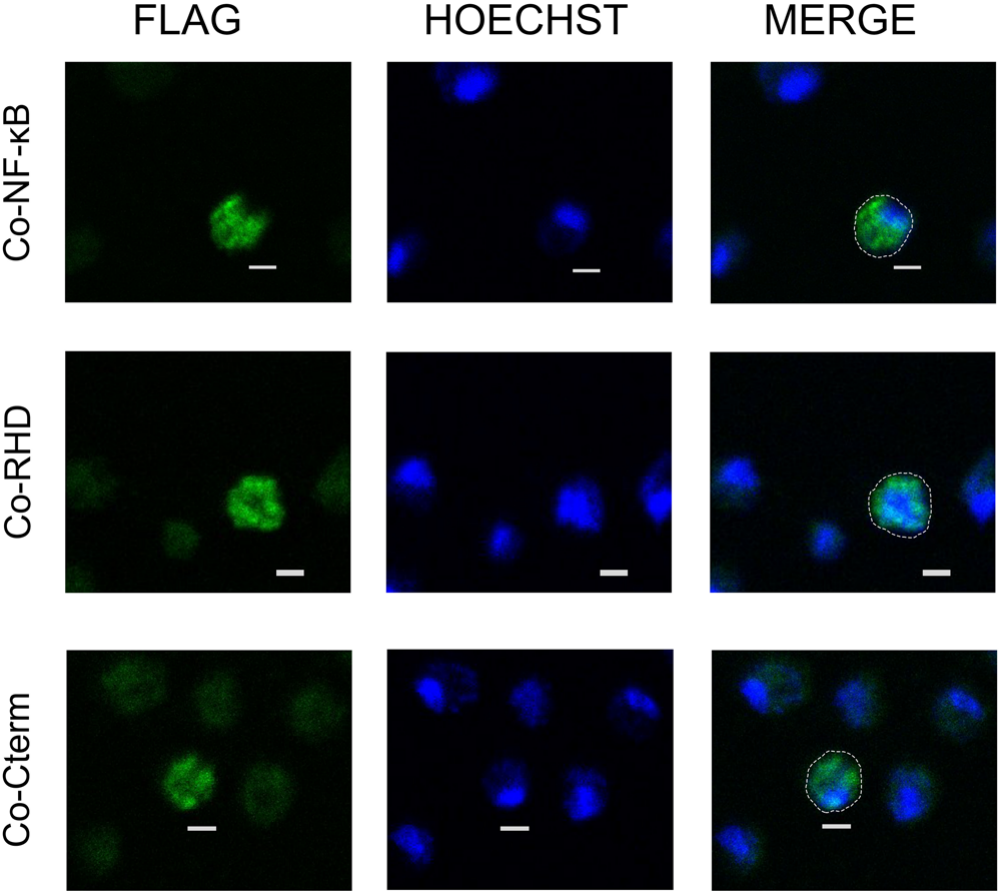
Transfection of FLAG-*Co*-NF-κB, FLAG-*Co*-RHD, and FLAG-*Co*-Cterm into *Capsaspora* cells results in cytoplasmic localization. *Capsaspora* cells were transfected with FLAG-tagged vectors for full-length *Co*-NF-κB, mutant *Co*-RHD, and mutant *Co*-Cterm. The cells were stained using anti-FLAG antiserum (left panels) and Hoechst (middle panels), and then merged (right panels). Scale bars are 2 µm. The dotted white lines indicate the periphery of the cells.

We were next interested in examining the subcellular localization of NF-κB in *Capsaspora* cells. For these experiments, we transfected *Capsaspora* cells with our FLAG-tagged*Co*-NF-κB constructs (*Co*-NF-κB, mutant *Co*-RHD, and mutant *Co*-Cterm, see Fig. 2a) and then performed anti-FLAG indirect immunofluorescence. Consistent with results seen in DF-1 chicken cells (Fig. 2d), FLAG-*Co*-NF-κB and FLAG-*Co*-Cterm localized to the cytoplasm of *Capsaspora* cells (Fig. 4, top and bottom rows). Surprisingly, FLAG-*Co*-RHD—which is nuclear in DF-1 cells—appeared to be primarily cytoplasmic in *Capsaspora* cells, as judged by its exclusion from the Hoechst-stained nuclei (Fig. 4, middle row).

### *Co*-NF-κB mRNA levels and DNA-binding activity vary coordinately across different life stages and the identification of putative *Co*-NF-κB target genes

*Capsaspora* has been shown to have three different life stages: aggregative, filopodia, and cystic, and RNA-Seq of each life stage has been reported^16^. We were interested in determining whether *Co-*NF-κB was active at different levels in these three life stages and whether we could use protein-binding microarray (PBM) data and the previous RNA-Seq data to identify genes whose expression may be controlled by NF-κB.

**Fig. 5:**
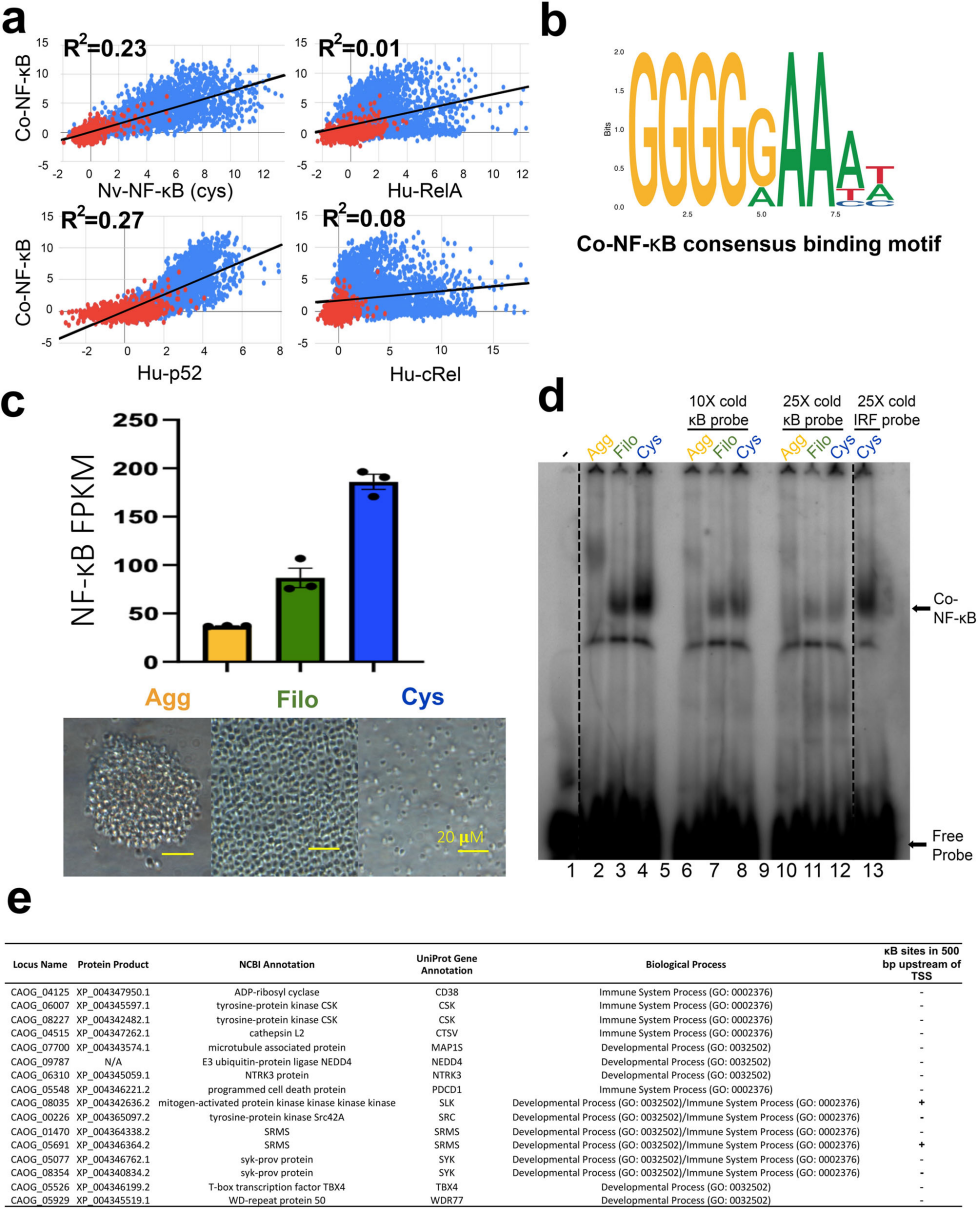
NF-κB is differentially expressed during the different life stages of *Capsaspora* and has possible target genes involved in development and immunity. **a** Protein binding microarray (PBM) DNA-binding profiles of *Co*-NF-κB as compared to *Nematostella vectensis* (*Nv*) NF-κB cysteine (cys) allele (top, left), human (Hu) RelA (top, right), human p52 (bottom, left), and human cRel (bottom, right). The axes are z-scores. Red dots represent random background sequences (*n* = 1159), and blue dots represent NF-κB binding sites (*n* = 2592). Black line is the best fit line (Co-NF-κB vs. the following: Nv-NF-κB, *R*^2^ = 0.23; Hu-p52, *R*^2^ = 0.27; Hu-cRel, *R*^2^ = 0.08; Hu-RelA, *R*^2^ = 0.01). **b** The consensus DNA-binding motif of *Co*-NF-κB generated from the PBM data in **a**. The motif was generated using the MEME motif discovery package^42^ using the 25 highest scoring binding sites identified by the PBM experiment in **a**. **c** Top: The FPKM values from Sebé-Pedrós et al.^16^ of NF-κB at each life stage, done in triplicate. Agg, Aggregative (yellow), Filo, Filopodic (green), Cys, Cystic (blue). Error bars are standard deviation. Bottom: Images taken with a light microscope of each life stage (Agg, Filo, and Cys from left to right). Yellow scale bar is 20 µm. Raw data are in Supplementary Data 5. **d** *Capsaspora* whole-cell extracts were created from each life stage (see Methods). 70 µg of each extract was then used in an electromobility shift assay, a palindromic κB-site probe (GGGAATTCCC). Lane 1 contains only free probe (-). Lanes 2-4 contain lysates from Agg, Filo, and Cys life stages incubated with a radioactive κB-site probe. Lanes 6–8, and lanes 10–12 contain lysates from Agg, Filo, and Cys life stages as indicated, and were incubated with an excess (10× and 25×, respectively) of unlabeled κB-site probe. Lane 13 contains the Cys lysate incubated with 25× unlabeled IRF-site probe. Lanes 5 and 9 contain no samples. NF-κB complexes and free probe are indicated with arrows. The dashed lines indicate where the image was cut to remove excess lanes. Raw image is in Supplementary Fig. 12. **e** The expression profiles of the indicated genes correlate with NF-κB mRNA expression in each life stage (Agg, low; Filo, medium; Cys, high), and were identified as developmental and immune system genes via Biological Processes GO analysis. Two of the genes in this list (*SRMS* and *SLK*) also contain κB sites in the 500 bp upstream of their TSS.

To determine the overall DNA binding-site specificity of *Co-*NF-κB, we analyzed a bacterially expressed *Co*-NF-κB RHD-only protein on a PBM containing 2592 κB-like sites and 1159 random background sequences (for array probe sequences, see ref. ^24^). By comparison of the z-scores for binding to DNA sites, the PBM-based DNA-binding profile of *Co-*NF-κB is most similar to NF-κBs from the sea anemone *N. vectensis* and human p52, and it is distinct from human cRel and RelA (Fig. 5a; (Co-NF-κB vs.: Nv-NF-κB, *R*^2^ = 0.23; Hu-p52, *R*^2^ = 0.27; Hu-RelA, *R*^2^ = 0.01; Hu-cRel, *R*^2^ = 0.08), which is consistent with the sequence and structural data indicating that *Co*-NF-κB is more like NF-κB proteins than Rel proteins. Based on these PBM data, we generated a DNA-binding motif logo for *Co*-NF-κB (Fig. 5b), which is quite similar to the binding motif of the p50:p50 homodimer^27^.

By examining previous mRNA expression data^16^ for NF-κB mRNA, we found that NF-κB mRNA was expressed at the lowest level in the aggregative stage and at 2.3- and 5-fold higher levels in the filopodic and cystic stages, respectively (Fig. 5c). We next generated cultures of *Capsaspora* at each life stage (Fig. 5c), made protein extracts, and performed an EMSA using the κB-site probe that we showed can be bound by *Co*-NF-κB expressed in HEK 293T cells (see Fig. 2e) and by bacterially expressed *Co*-RHD in our PBM assays (Supplementary Data 1). Consistent with the mRNA expression data, the κB-site probe was bound progressively stronger in lysates from aggregative, filopodic, and cystic stage cells (Fig. 5d). To confirm that the EMSA band included *Co*-NF-κB, we incubated our protein extracts with 10- and 25-fold excesses of unlabeled κB-site probe, and we saw a substantial decrease in binding in the putative NF-κB band. In contrast, incubation with a 25-fold excess of an unlabeled IRF-site probe did not decrease the putative *Co*-NF-κB complex, indicating that the binding activity in the extracts was specific for the κB-site probe (Fig. 5d).

We next sought to identify genes that might be influenced by the expression of NF-κB in these life stages. For this analysis, we examined existing RNA-Seq data^16^, which contains mRNA expression data for 8674 genes of *Capsaspora* during its three life stages. We first narrowed our gene list to those genes that are differentially expressed in a manner similar to NF-κB mRNA levels and DNA-binding activity during each life stage (i.e., genes that are progressively increased in expression in aggregative, filopodic, and cystic stages). From this exercise, we identified 1348 mRNAs that were expressed at low levels in the aggregative stage, and progressively higher levels in the filopodic and cystic stages (Supplementary Data 2).

Of the 1348 genes that we identified, 389 genes were annotated (which is consistent with approximately 25% of *Capsaspora*’s total predicted protein-encoding genes being annotated^4,16^), and 305 of these 389 genes had human homologs (Supplementary Data 2). We then performed GO analysis to identify biological processes overrepresented in these 305 genes. The GO analysis showed that this set of 305 genes was enriched for genes predicted to be involved in several biological processes (Supplementary Fig. 4), including 16 *Capsaspora* genes that encode proteins associated with developmental and immune system processes (Fig. 5e), which are biological processes regulated by NF-κB in many multicellular organisms and suggested to be regulated by NF-κB in several early-branching organisms^22–25,28–30^. Although 16 genes may seem low, the total database for human GO analysis of immune system and developmental processes genes is approximately 2200 genes, but the number of annotated homologs that exist in *Capsaspora* in these two GO categories is only 66 genes (Supplementary Table 3). Thus, about 25% (16/66) of the *Capsaspora* genes in the GO category for the developmental and immune processes subset are among the 305 homologous genes coordinately expressed with NF-κB. Other broad GO categories overrepresented in these 305 genes included Signaling, Metabolic Process, and Locomotion (Supplementary Fig. 4).

We then looked for κB sites within 500 base pairs (bp) upstream of the transcription start sites (TSS) for each of the 1348 genes with expression profiles that were similar to NF-κB using the *Co*-NF-κB DNA-binding motif (Fig. 5b) generated from the PBM analysis. The upstream regions of 235 of these 1348 genes contained 1-4 κB sites within 500 bp of the TSS, with the majority of these genes containing 1 κB site (Supplementary Data 3). Two (*SRMS* and *SLK*,) of the 16 genes that are coordinately regulated with *Co*-NF-κB and encode protein homologs associated with GO developmental and immune system processes also contain a κB site within the 500 bp upstream of their TSS (Fig. 5e). Of note, one gene (*5′-AMP-activated protein kinase catalytic subunit alpha-2*) contains four upstream κB sites and six genes have three κB sites within 500 bp upstream of their TSS (Supplementary Data 3).

### Choanoflagellate NF-κBs can form heterodimers and have different abilities to bind DNA and activate transcription

**Fig. 6:**
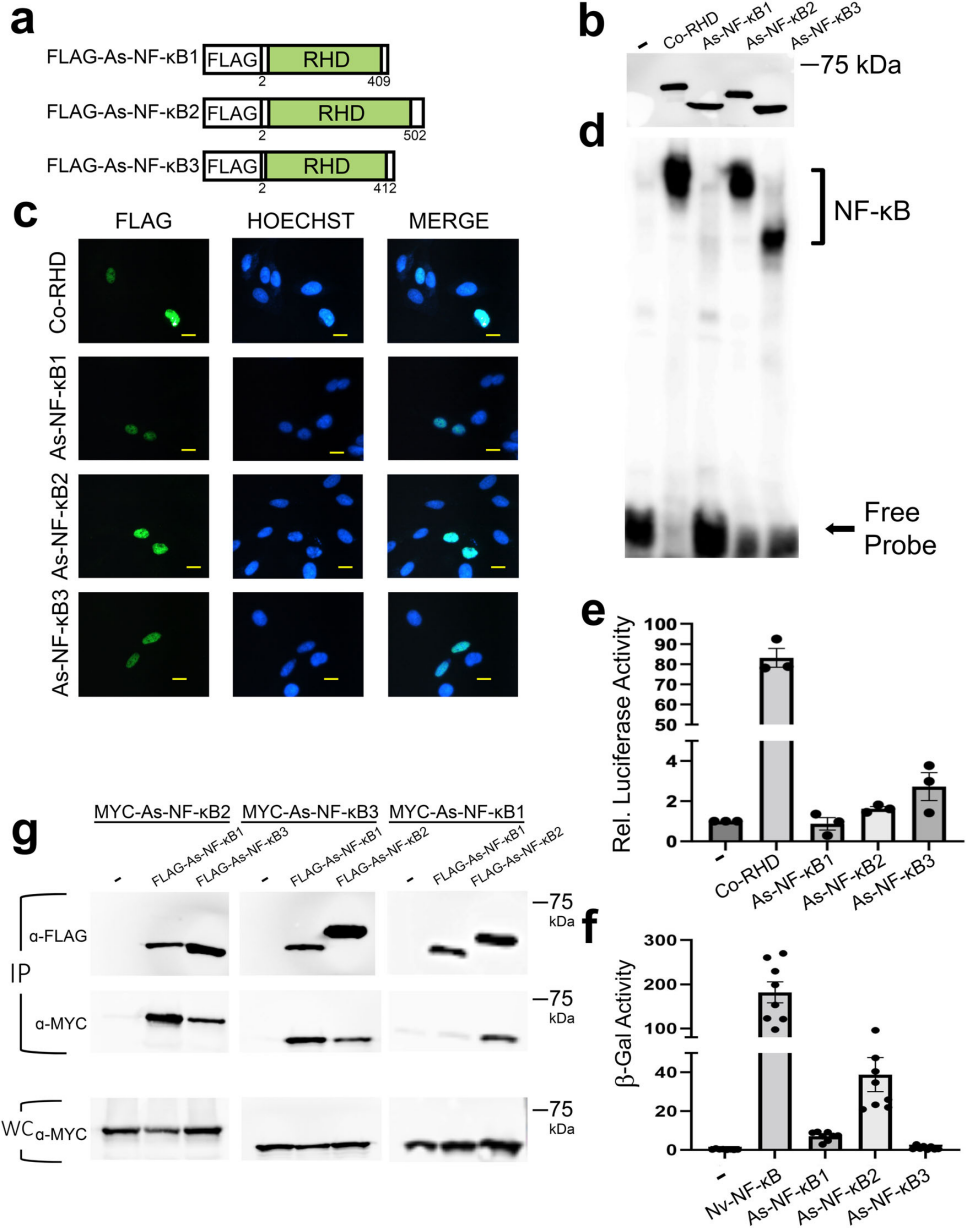
Characterization of cellular and molecular properties of three choanoflagellate NF-κBs. **a** FLAG-tagged NF-κB proteins used in these experiments. From top to bottom the drawings depict the three NF-κB-like proteins from the transcriptome of *A. spectabilis*^5^. RHDs are in green (Rel Homology Domain). **b** Anti-FLAG Western blot of lysates from HEK 293T cells transfected with the indicated expression vectors or the vector control (-). Raw image is in Supplementary Fig. 13. **c** Indirect immunofluorescence of DF-1 chicken fibroblasts transfected with the indicated expression vectors. Cells were then stained with anti-FLAG antiserum (left panels) and Hoechst (middle panels), and then merged (right panels). Yellow scale bar is 10 µm. **d** A κB-site electromobility shift assay using a palindromic κB-site probe (GGGAATTCCC) and each of the indicated lysates from **b**. The NF-κB complexes and free probe are indicated by arrows. Raw image is in Supplementary Fig. 13. **e** A κB-site luciferase reporter gene assay was performed with expression vectors for the indicated proteins or the empty vector control (-) in HEK 293 cells. Luciferase activity is relative (Rel.) to that seen with the empty vector control (1.0). Values are averages of *n* = 3 biological replicates per sample each performed in triplicate, and are reported with standard error. Raw data are in Supplementary Data 5. **f** A GAL4-site *LacZ* reporter gene assay was performed with the indicated GAL4-fusion proteins or the GAL4 alone vector control (-) in yeast Y190 cells. Values are average β-gal units of *n* = 8 biological replicates. Raw data are in Supplementary Data 5. **g** Co-immunoprecipitation (IP) assays of MYC-tagged *As*-NF-κB1, *As*-NF-κB2, *As*-NF-κB3. In each IP assay, MYC-*As*-NF-κBs were co-transfected in HEK 293T cells with the pcDNA-FLAG vector control (-), or FLAG-*As*-NF-κB1, 2 or 3 as indicated. An IP using anti-FLAG beads was performed, and pulled down proteins were then analyzed by Western blotting with anti-FLAG (top) or anti-MYC (middle) antisera. An anti-MYC Western blot of 5% of the whole-cell (WC) lysates used in the pulldowns was also performed (bottom). Raw image is in Supplementary Fig. 13.

Richter et al.^5^ identified transcripts encoding RHD-containingNF-κB-related proteins in 12 choanoflagellate species. We chose to characterize the NF-κB proteins from *Acanthoeca spectabilis* (*As*) because it has three NF-κB-like proteins, which separated into multiple branches when phylogenetically compared to other choanoflagellate NF-κBs (Supplementary Fig. 1b). The three *As*-NF-κB proteins contain ostensibly complete DNA-binding sequences, which are similar to other NF-κB proteins, and a putative NLS^3^. These three *As*-NF-κB proteins contain few residues C-terminal to the RHD (and no GRRs or ANK repeats). As a first step in characterizing these proteins, we created pcDNA-FLAG vectors for *As*-NF-κB1, *As*-NF-κB2, and *As*-NF-κB3 (Fig. 6a) and transfected them into HEK 293T cells. As judged by anti-FLAG Western blotting, each plasmid directed the expression of an appropriately sized FLAG-tagged*As*-NF-κB protein (Fig. 6b, Supplementary Table 1).

To determine the subcellular localization properties of the three *As*-NF-κBs, we performed indirect immunofluorescence using DF-1 chicken cells transfected with each FLAG-tagged vector. All three *As*-NF-κB proteins co-localized with Hoechst-stained nuclei in DF-1 cells (Fig. 6c).

We then performed a κB-site EMSA on whole-cell extracts from HEK 293T cells overexpressing each *As*-NF-κB, using *Co*-RHD as a positive control. *As*-NF-κB2 and 3 bound the κB-site probe to nearly the same extent as *Co*-RHD, but *As*-NF-κB1 only weakly bound the probe (Fig. 6d). We also assessed the transactivating ability of each *As-*NF-κB protein in a κB-site reporter assay in 293 cells, using the strongly activating *Co*-RHD protein as a positive control. Both *As*-NF-κB2 and 3 were able to activate transcription of the luciferase reporter above vector control levels (~1.6- and 2.7-fold, respectively), but *As*-NF-κB1 did not (Fig. 6e). We also determined the intrinsic transactivating ability of each *As*-NF-κB protein using a GAL4 DNA-binding domain-fusion protein reporter assay in yeast cells. In this assay, all three *As*-NF-κBs activated transcription above the GAL4 alone vector control levels, although *As*-NF-κB1 and 3 activated to a much lesser extent than *As*-NF-κB2 (Fig. 6f). From these data, we hypothesized that the homodimeric version of *As*-NF-κB1 was not capable of binding DNA and therefore, would not be expected to activate transcription of a κB-site reporter, but likely contained some intrinsic ability to activate transcription (based on its ability to activate transcription as a GAL4-fusion protein in yeast). In contrast, homodimeric *As*-NF-κB2 and 3 could both activate transcription in human cell-based κB-site and yeast GAL4-fusion protein reporter assays.

Since *As*-NF-κB1 did not substantially bind DNA or activate transcription when expressed alone in 293 cells, we hypothesized that *As*-NF-κB1 normally only acts as a heterodimer with the other *As*-NF-κBs. To determine whether *As*-NF-κB1 could interact with itself or *As*-NF-κB2 or 3, we performed a series of co-immunoprecipitation experiments (Fig. 6g). To do this, we subcloned *As*-NF-κB1, 2, and 3 into MYC-tagged vectors, co-transfected each with FLAG-*As*-NF-κB1 (as well as FLAG-*As*-NF-κB2 and 3) into 293T cells, and first immunoprecipitated FLAG-*As*-NF-κB1 from cell extracts using anti-FLAG beads. We then performed anti-FLAG and anti-MYC Western blotting on the immunoprecipitates to determine whether these NF-κBs could interact. MYC-*As*-NF-κB2 and MYC-*As*-NF-κB3 were both co-immunoprecipitated with FLAG-*As*-NF-κB1, as well as with FLAG-tagged versions of themselves (Fig. 6g, IP). In contrast, MYC-*As*-NF-κB1 was not co-immunoprecipitated with the FLAG-tagged version of itself. The MYC-*As*-NF-κB proteins were not seen in anti-FLAG immunoprecipitates when they were co-transfected with the empty vector control (Fig. 6g, IP).

From these data, it appears that all three *As-*NF-κBs can enter the nucleus when expressed in vertebrate cells, but they bind DNA and activate transcription to varying degrees. Of note, the transcriptional activating abilities of the three *As*-NF-κB proteins, both from a κB-site reporter in 293 cells and from a GAL4-site reporter in yeast, are not nearly as strong as the *Co*-RHD proteins (compare Figs. 2 and 6). Furthermore, all three *As*-NF-κBs can form heterodimers with the other *As*-NF-κBs, but *As*-NF-κB1 cannot form a homodimer. Therefore, it is likely that the limited ability of *As*-NF-κB1 to bind DNA and activate a κB-site reporter gene is due to its inability to efficiently form homodimers.

## Discussion

In this manuscript, we have functionally characterized and compared NF-κB proteins from two protists. Our results demonstrate that functional DNA-binding and transcriptional-activating NF-κB proteins exist in both of these protists, but the overall structures, activities, and regulation of these proteins vary considerably, both among protists and when compared to animal NF-κBs.

In the vertebrate NF-κB proteins p100 and p105, C-terminal ANK repeats inhibit the DNA-binding activity of the RHD. IKK-induced proteasome-mediated processing of the ANK repeats is terminated within the GRR^21^, and this processing activates the DNA-binding activity of these mammalian NF-κB proteins. In the *Drosophila* Relish protein, the C-terminal ANK repeats also inhibit RHD DNA binding, but Relish has no GRR and the C-terminal ANK repeats are removed by an internal site-specific proteolytic cleavage event, which does not involve the proteasome^31^. Thus, the presence of ANK repeats and a GRR in *Co*-NF-κB suggests that proteasomal processing would lead to nuclear translocation and activation of its DNA-binding activity. Indeed, removal of the C-terminal residues of *Co*-NF-κB does allow it to enter the nucleus of vertebrate cells and unleashes its DNA-binding and transactivation activities (Fig. 2c–h). Moreover, the proteasome inhibitor MG132 blocks the basal processing of *Co*-NF-κB that is seen in transfected 293T cells (Supplementary Fig. 3b). However, this basal processing of *Co*-NF-κB in 293T cells is not enhanced by co-expression of IKK (unless C-terminal IKK target sequences are added, see Fig. 3b) and there are no obvious IKK target serines in *Co*-NF-κB nor are there any IKK homologs in the *Capsaspora* genome. If *Co*-NF-κB does undergo a signal-induced processing in *Capsaspora* cells, it is unlikely to be dependent on an IKK-like kinase.

Using proteomic analyses, Sebé-Pedrós et al.^17^ showed that *Co*-NF-κB protein levels and the phosphorylation of two C-terminal serines are low in aggregative stage cells and higher in cystic stage cells. This pattern of protein expression and phosphorylation of *Co*-NF-κB parallels its mRNA expression (Fig. 5c) and DNA-binding activity (Fig. 5d). Thus, phosphorylation of the two C-terminal serines identified by phosphoproteomic analysis may be involved in activation of the DNA-binding activity of *Co*-NF-κB or may simply reflect increased amounts of *Co*-NF-κB in aggregative vs cystic cells. The presence of increased levels of C-terminal*Co*-NF-κB residues in cystic cells, as judged by proteomic analysis^17^, indicates that these sequences are intact in this stage, which has high NF-κB DNA-binding activity. In *Drosophila*, the C-terminal sequences of Relish are not degraded after signal-induced cleavage^31^. Whether the C-terminal sequences of *Co*-NF-κB identified by proteomics are part of the full-length protein or have been cleaved but are still intact in native *Capsaspora* cells is not known. *Co*-NF-κB-specific antiserum would be required to address this question.

In contrast to *Capsaspora*, the choanoflagellate NF-κB proteins lack C-terminal ANK repeats and GRRs, and all three *As*-NF-κB proteins are constitutively in the nucleus when overexpressed in vertebrate cells (Fig. 6c). We have not been able to identify an IκB-like protein in the *A. spectabilis* transcriptome. Thus, it is unclear whether choanoflagellate NF-κB is regulated by an ANK-dependent mechanism, or whether, for example, choanoflagellate proteins are constitutively nuclear in their native settings.

Constitutively nuclear localization of NF-κB proteins has also been seen in other settings. That is, we have previously shown that in both the sea anemone *Aiptasia* and one sponge most NF-κB staining is nuclear, and these NF-κB proteins are mostly processed in their native settings^22–24^. Thus, we have argued previously^3^ that NF-κB proteins in these early-branching animals may be constitutively in an active state, perhaps due to continual stimulation by upstream activating ligands or pathogens. Of note, most NF-κB p100 is also in its processed form in mouse spleen tissue^32^.

Among the 21 choanoflagellates for which there is sufficient transcriptomic/genomic information, only 12 have been found to have NF-κB genes^5,19,20^. In seven of these 12 choanoflagellates there are multiple NF-κB genes^5^. Thus, it appears that there have been gains and losses of NF-κB genes among the choanoflagellates. We note that NF-κB has also been lost in other organisms including *C. elegans* and ctenophores^3^. The apparent absence of NF-κB in some choanoflagellates and its expansion in others (e.g., *A. spectabilis*) suggest that NF-κB has a specialized, rather than a general and required, biological function in choanoflagellates.

The presence of three *A. spectabilis*NF-κB-like proteins that can form heterodimers is the only example of an organism in a lineage predating flies that has multiple NF-κB proteins which are capable of forming heterodimers. Thus, expansion of NF-κB genes has occurred at least two times in evolution, i.e., at least once in the metazoan lineage and once within choanoflagellates. Furthermore, since each *As*-NF-κB homodimer has a different ability to form homodimers, bind DNA, and activate transcription, it appears that there are subclasses of NF-κB dimers in *A. spectabilis* and likely in other choanoflagellates that have multiple NF-κBs. It is interesting to note that *As*-NF-κB1, 2, and 3 phylogenetically separate and cluster most closely with NF-κBs from certain other choanoflagellate species that contain multiple NF-κBs (Supplementary Fig. 1b and ref. ^3^). For example, *Savillea parva* contains three NF-κBs, each of which clusters with a separate *As*-NF-κB (Supplementary Fig. 1b and ref. ^3^). Thus, we hypothesize that choanoflagellates, like vertebrates, have evolved a mechanism for differential transcriptional control of genes through the use of combinatorial NF-κB dimer formation.

In both NF-κB-site reporter assays in human 293 cells and in GAL4-site reporter assays in yeast, *Co*-RHD is more strongly activating than the individual *As*-NF-κB proteins (i.e., compare data from Fig. 2e, f to Fig. 6e, f). These two reporter assays are, of course, measuring different activities—NF-κB site-based activation vs. intrinsic activation when fused to the GAL4 DNA-binding domain—and in non-native cell types (human and yeast cells, respectively). *As*-NF-κB1 does not activate from the NF-κB-site reporter (Fig. 6e) because it does not bind to DNA (Fig. 6d); however, it can activate if brought to DNA by the GAL4 DNA-binding domain (Fig. 6f). In contrast *As*-NF-κB-3 (competent for DNA-binding [Fig. 6d]) can activate the NF-κB-site reporter (Fig. 6e), but when bound to GAL4 *As*-NF-κB-3 does not have substantial transactivation ability (Fig. 6f). These results suggest that heterodimers (e.g., between *As*-NF-κB-1 and *As*-NF-κB-3) are relevant dimers in vivo in their native organism. It will be interesting to assess the NF-κB-site transactivating abilities of *As*-NF-κB heterodimers, especially given that heterodimers are the most relevant dimer complexes in mammals^1,2^.

The differential mRNA expression and DNA-binding activity of NF-κB among different life stages of *Capsaspora* suggest that *Co*-NF-κB has life stage-specific functions. It is notable that the DNA-binding activity of NF-κB in these different life stages correlates with differences in the levels of NF-κB mRNA^16^ and protein^17^, rather than as differences in induced DNA-binding activity. In most metazoans, the activity of NF-κB is regulated at the post-transcriptional level, whereas in *Aiptasia* and corals, we have found that the levels of NF-κB mRNA, protein, and DNA-binding activity appear to be coordinately regulated, suggesting that the regulation of NF-κB occurs at the transcriptional level. For example, in *Aiptasia*, thermal bleaching causes transcriptional upregulation of NF-κB, which also results in increased protein expression of nuclear, DNA binding-active NF-κB^24^. Similarly, treatment of the coral *Orbicella faveolata* with lipopolysaccharide results in increased expression of NF-κB pathway genes, rather than increased post-translational activation of NF-κB^22^. Since there has yet to be an IκB-like homolog identified in choanoflagellates, it is possible that the activity of the choanoflagellate NF-κB proteins is fully regulated by transcriptional control of their genes. Thus, it appears that induced activity of NF-κB in several early-branching organisms is the result of transcriptional upregulation of NF-κB mRNA, rather than induced proteolysis, which occurs in most mammalian and fly systems.

Of the nearly 1350 genes whose expression correlated with NF-κB expression across different *Capsaspora* life stages, almost 20% contained predicted *Co*-NF-κB binding sites within 500 base pairs upstream of their TSS (Supplementary Data 3). While 20% is almost certainly an overestimate of *Co*-NF-κB direct or indirect gene targets, there are also likely additional NF-κB binding sites that could affect target gene expression. For example, ATAC-seq data suggested that the regulatory sites in the *Capsaspora* genome are present in first introns, 5′ UTRs, as well as the proximal intergenic regions^33^.

The list of ~1350 genes that are coordinately expressed with *Co*-NF-κB mRNA most certainly contains genes that are controlled by other transcription factors or are regulated by signaling or developmental events that do not involve NF-κB. Additionally, within this gene set, there are enriched GO terms other than immune system and development such as multi-organism process, interspecies interaction between organisms, and multicellular organism process. One could speculate that aggregation in *Capsaspora* and the correlative decrease in NF-κB in aggregated cells reflect a need to suppress collective immunity to form a symbiotic group. Alternatively, given that *Capsaspora* is normally a symbiont in the hemolymph of the snail *B. glabrata*^9,14,15^, NF-κB may play a role in maintaining symbiosis, which has been suggested as one function of NF-κB in other organisms^34^. In any event, it is clear that NF-κB may play several roles, and perhaps different roles, in each life stage of *Capsaspora*.

It is not clear what type of pathway controls activation of NF-κB in protists. In early-branching organisms, many common upstream NF-κB-activating components are missing, few in number, or lack critical domains (Supplementary Fig. 5). For example, *Capsaspora* does not contain homologs to TLRs, ILR-1 or TNFRs^4^. However, choanoflagellates do contain both full-length TLR-like and TIR-only homologs, as well as cGAS and STING homologs^5^, which mediate innate immune responses in many organisms.

The differences in NF-κB that we describe here between *Capsaspora* and choanoflagellates, which are members of the same group (Opisthokont) of protists, suggest that the diversification of NF-κB among all protists is considerable. The continued study of the evolution of NF-κB and other basally derived transcription factors will likely lead to an understanding of where and how these factors originated, as well as the basal biological functions they control.

## Methods

### Phylogenetic analyses

For Supplementary Fig. 1, the RHD sequences of NF-κB from *C. owczarzaki* and choanoflagellates were compared to the NF-κB-like sequences present in several other organisms. Details on databases and sequence acquisition can be found in Supplementary Data 4. For the phylogenetic analysis in Supplementary Fig 1b, sequences were aligned by Clustal Omega^35^. A maximum likelihood phylogenetic tree was created using www.phylogeny.fr in one-click mode^36^, rooted to *Capsaspora*, and is expanded on in ref. ^3^.

### Plasmids

Human cell codon-optimized versions of *Co*-NF-κB and the three *As*-NF-κBs were synthesized by GenScript and were provided as cDNA subclones in pUC57-Simple; their sequences are shown in Supplementary Figs. 6–9. Plasmids of the following types were used in these experiments: (1) pcDNA-based plasmids for the expression of epitope-tagged (FLAG, HA, MYC) proteins in tissue culture and *Capsaspora* cells; (2) pGBT9-based plasmids for the expression of GAL4 (aa 1–147) fusion proteins in yeast; (3) p3x-κB-luc reporter plasmid that has three NF-κB binding sites upstream of the luciferase reporter gene for transcriptional reporter gene assays in HEK 293 cells. Details of primers used for plasmid constructions (Supplementary Table 4) and plasmid sources and plasmid construction strategies (Supplementary Table 5) are provided. All plasmids were purified with a Midiprep kit (ZymoPURE, #D413) prior to use.

### Cell culture and transfection

DF-1 immortalized chicken fibroblasts (Douglas Foster, University of Minnesota)^37^ and human HEK 293 and 293T cells (ATCC) were grown at 37 °C/5% CO_2_ in Dulbecco’s modified Eagle’s Medium (DMEM) (Invitrogen) supplemented with 10% fetal bovine serum (FBS) (Biologos), 50 units/ml penicillin, and 50 μg/ml streptomycin. *Capsaspora* cell cultures (strain ATCC ®30864) were grown axenically in 25 cm^2^ culture flasks (ThermoScientific Nunclon) with 10 ml ATCC medium 1034 (modified PYNFH medium) at ~21 °C. Transfection of cells with expression plasmids was performed using polyethylenimine (PEI) (Polysciences, Inc., #23966).

HEK 293T cells were transfected for the expression of epitope-tagged proteins from pcDNA-based plasmids. These cells were used because they stably express the large T-antigen of SV-40, which enables high-level expression of transfected plasmids from plasmids (like pcDNA) that contain the SV-40 origin of replication. For transfection, cells were plated out in 60-mm tissue culture plates at approximately 60–80% confluence. The next day, cells were transfected as follows: (1) 5 μg of pcDNA expression plasmid and 30 μl of 1 mg/ml PEI were mixed in 300 μl of DMEM (no serum or antibiotics); (2) samples were incubated for 15–20 min at room temperature with occasional swirling: (3) 1.5 ml of DMEM/10% FBS was added to the transfection mix; and (4) the transfection mix was added to the cells after removing the media from the cells. The next day, the media was removed, and replaced with fresh DMEM/10% FBS. The following day, cell lysates were prepared in AT Lysis Buffer as described below.

Chicken DF-1 fibroblast cells were used for indirect immunofluorescence of over-expressed proteins because of their flat, easy-to-visualize morphology. DF-1 cells were plated in DMEM containing 10% FBS at about 40% confluence in a 35-mm tissue culture dish. The next day, cells were transfected with the indicated pcDNA vector as follows: (1) 3 μg of plasmid DNA and 18 μl of 1 mg/ml PEI were added to 80 μl of DMEM (no serum); (2) the mix was incubated for 15–20 min at room temperature with occasional swirling; (3) 2 ml DMEM/10% FBS was then added to the transfection mix; and (4) the mix was added to the cells in the 35-mm dish (after removing the media on the plate). Approximately 20 h after adding the DNA, the transfection media was removed and fresh DMEM/10% FBS was added. After another 24 h, cells were passed onto UV-sterilized coverslips at about 60% confluence (with coverslips in a 35-mm tissue culture dish). Cells were grown overnight at 37 °C/5% CO_2_ in a tissue culture incubator.

*Capsaspora* cells in culture were transfected with pcDNA-FLAG expression plasmids for analysis by indirect immunofluorescence. The day before transfection, actively dividing *Capsaspora* filopodic cells were plated at 80-90% confluency in 35-mm tissue culture dishes. The next day, cells were transfected. This was carried out by first preparing a transfection mix as follows: (1) 5 μg of pcDNA expression plasmid and 25 ul of 1 mg/ml PEI was added to 300 μl of PBS; (2) the mix was incubated for 15–20 min at room temperature with occasional swirling; (3) 2.2 ml of *Capsaspora* media (ATCC medium 1034) was then added to the mix; and media was removed from the 35-mm dish containing cells, and the transfection mix was added to the plate. Media was changed ~20 h post-transfection. Approximately 24 h later, *Capsaspora* cells to be analyzed by immunofluorescence were passaged onto poly-L-lysine (Sigma, #P4707)-treated glass coverslips one day prior to fixation for immunofluorescence processing.

### Generation of *Capsaspora* cells at three life stages

Cultures for each *Capsaspora* life stage were generated according to Sebé-Pedrós et al.^16^ and as instructed by ATCC. That is, filopodic cells were maintained in an actively dividing adherent state by scraping and passaging 1/40-1/50 of the cultures every 6–8 days, before floating cells appeared. Floating cystic cells were collected from 14 day-old filopodic cultures. Aggregative cells were created by actively scraping dividing filopodic cells and seeding them into a 25 cm^2^ culture flask, which was gently agitated at 60 RPM for 4–5 days, and grown axenically at ~21 °C. For EMSAs, whole-cell lysates of cells from each life stage were prepared in AT Lysis Buffer as described below.

### Preparation of cell lysates for Western blotting and EMSAs

Whole-cell protein lysates of transfected HEK 293T cells in 60-mm plates were made for Western blotting and for electrophoretic mobility shift assays. Generally, cells were harvested 48 h post-transfection. Tissue culture plates containing HEK 293T cells were placed on ice, and washed gently two times with PBS. Cells were then removed with a cell scraper into PBS, transferred to a 1.5-ml tube, and cells were collected by centrifugation at 3,000 rpm for 5 min. Cells were then resuspended in 1 ml of PBS, pelleted at 3,000 rpm for 5 min, and the PBS was removed by aspiration. Cells were resuspended by pipetting in 100 μl of AT Lysis Buffer (20 mM HEPES, pH 7.9, 1 mM EDTA, 1 mM EGTA, 20% wt/vol glycerol, 1% w/v Triton X-100, 20 mM NaF, 1 mM Na_4_P_2_O_7_·10H_2_O, 1 mM dithiothreitol, 1 mM phenylmethylsulfonyl fluoride, 1 μg/ml leupeptin, 1 μg/ml pepstatin A, 10 μg/ml aprotinin). Cell samples were then passed five times through a 27.5- gauge needle to ensure complete lysis. NaCl was then added to a final concentration of 150 mM and protein lysates were clarified by centrifugation at 13,000 rpm for 25 min at 4 °C. Supernatants were then transferred to fresh 1.5-ml microcentrifuge tubes and stored at −80 °C. Protein concentration was determined using the Bio-Rad Protein Assay dye reagent (Bio-Rad, Cat# 5000006).

Whole-cell lysates of *Capsaspora* cells from each life stage (see below) were prepared for EMSAs. On the day of lysis, cells for each cell stage from five, 25 cm^2^ culture flasks (ThermoScientific Nunclon) were gently washed once with ice-cold PBS and were then removed with a rubber scraper into 1 ml of PBS for each plate and transferred into five, 1.5-ml microcentrifuge tubes. The cells were pelleted at 3000 rpm for 5 min at 4 °C and then combined and resuspended by pipetting in 250 ul of AT Lysis Buffer (see above). Samples were then passed five times through a 27.5-gauge needle to ensure complete lysis. NaCl was then added to a final concentration of 150 mM and protein lysates were clarified by centrifugation at 13,000 rpm for 25 min at 4 °C. Supernatants were then transferred to fresh 1.5-ml microcentrifuge tubes and stored at −80 °C. Protein concentration was determined using the Bio-Rad Protein Assay dye reagent (Bio-Rad).

### Western blotting and electrophoretic mobility shift assays (EMSAs)

Western blotting was performed essentially as described in detail elsewhere^26,38^. Briefly, extracts (~50 μg) of transfected HEK 293T cells were resuspended in 2X SDS sample buffer (0.125 M Tris-HCl [pH 8.0}, 4.6% w/v SDS, 20% w/v glycerol, 10% v/v β-mercaptoethanol, 0.2% w/v bromophenol blue) and heated at 90 °C. Proteins were then separated on 7.5% or 10% SDS-polyacrylamide gels. Proteins were then transferred to nitrocellulose at 4 °C at 250 mA for 2 h followed by 170 mA overnight. The membrane was blocked in TBST (10 mM Tris-HCl [pH 7.4], 150 mM NaCl, 0.1% v/v Tween-20) containing 5% powdered non-fat milk (Carnation) for 1 h at room temperature. Filters were incubated at 4 °C with anti-FLAG primary antiserum (1:1000, Cell Signaling Technology, #2368) or anti-HA primary antiserum (1:500, Santa Cruz Biotechnology, #sc-805) diluted in TBST containing 5% non-fat powdered milk. After extensive washing in TBST, filters were incubated with anti-rabbit horseradish peroxidase-linked secondary antiserum (1: 4000, Cell Signaling Technology, #7074). Immunoreactive proteins were detected with SuperSignal West Dura Extended Duration Substrate (ThermoFisher, #34075) and imaged on a Sapphire Biomolecular Imager (Azure Biosystems).

We have recently described our procedures for EMSAs in detail^38^. DNA-binding reactions were performed in DNA-binding reaction buffer (10 mm HEPES [pH 7.8], 50 mM KCl, 1 mM DTT, 1 mM EDTA, 4% w/v glycerol, 40 μg/ml poly-dI-dC, 2X BSA) containing 200,000 cpm of a ^32^P-labeled κB-site double-stranded probe (GGGAATTCCC, see Supplementary Table 5) and whole-cell extracts from 293T (50 μg total protein) or *Capsaspora* cells (70 μg total protein) prepared in AT lysis buffer. Samples were incubated at 30 °C for 30 min. Samples were then separated on 5% non-denaturing polyacrylamide gels. After drying, gels were exposed to a phosphor screen, and then imaged on a Sapphire Biomolecular Imager (Azure Biosystems).

### Reporter gene assays

NF-κB-site luciferase reporter assays were performed in HEK 293 cells as these assays do not require the SV-40 T-antigen, which can also affect expression from some reporter plasmids. Co-transfections with 0.5 μg of 3× κB-site luciferase reporter plasmid and 2 μg of pcDNA-FLAG expression plasmids were performed in triplicate for each expression plasmid in 35-mm plates using 15 μg PEI as described above for HEK 293T cells. Two days after transfection, cells were lysed and luciferase activity was measured on lysates that contained equal amounts of protein using the Luciferase Assay System (Promega, #E397A). Luciferase activity for each triplicate were averaged and were normalized to the empty vector control (set as 1.0). Three independent experiments were performed (with triplicate samples), and values are reported as the averages of the three experiments plus standard error (SE), determined as follows:

1 $${{{{{\rm{SE}}}}}}=\sigma /\sqrt{{{{{{\rm{n}}}}}}}$$

where σ is the sample standard deviation, and n is the number of independent experiments.

For GAL4-fusion protein reporter gene assays, yeast strain Y190, which contains an integrated GAL4-site*lacZ* reporter locus, were used. Y190 cells were transformed with pGBT9-based plasmids and stable transformants were selected on plates containing yeast media but lacking tryptophan. Independent colonies were picked and grown overnight in 3 ml of liquid media lacking tryptophan. The density of the culture was measured by visible light spectrometry at OD_595_, as an indication of cell number. For reporter gene activity, 1.0 ml of cells was pelleted and cells were lysed by freeze-thawing five times in 90 μl of 100 mM Tris [pH 7.6], 0.05% Triton X-100. To each sample, 450 µl of Z/ONPG solution (60 mM Na_2_HPO_4_, 40 mM NaH_2_PO_4_, 10 mM KCl, 1 mM MgSO_4_, 50 mM 2-mercaptoethanol, 0.8 mg/ml ONPG [orthonitrophenyl-β-D-galactopyranoside; Sigma], 1 mM DTT, 0.005% SDS) was added. Samples were incubated at 30 °C until yellow color appeared or for a maximum of 30 min for the negative control. The reaction was terminated by the addition of 225 µl of 1 M Na_2_CO_3_. Samples were clarified by centrifugation at top-speed for 2 min in a microcentrifuge, and the amount of β-galactosidase activity was measuring at OD_415_. The *lacZ* reporter gene transactivation was determined by calculating the Units of β-galactosidase activity as follows:

2 $${{{{{\rm{Units}}}}}}=\frac {1000\times {{{{{{\rm{OD}}}}}}}_{415}}{{{{{{\rm{V}}}}}}\times {{{{{\rm{t}}}}}}\times {{{{{{\rm{OD}}}}}}}_{595}}$$

where *V* = volume of cells used (1.0 ml) and *t* = time (min) until the reaction was terminated.

Values for samples (number of samples indicated in figure legends) were averaged and are reported as average Units with SE (determined as in Eq. 1 above).

### Co-immunoprecipitations

HEK 293T cells in 60-mm dishes were co-transfected with 2.5 μg of MYC-*As*-NF-κB1, 2, or 3 and 2.5 μg of either a pcDNA-FLAG empty vector, FLAG-*As*-NF-κB1, 2 or 3 (Fig. 6g), or were co-transfected with 2.5 μg of MYC-*Co*-Cterm and 2.5 μg of either pcDNA-FLAG or FLAG-*Co*-RHD (Fig. 2g), using PEI as described above. Lysates were prepared 48 h later in AT Lysis Buffer and were incubated with 50 μl of a PBS-washed anti-FLAG bead 50% slurry (Sigma, #A2220) overnight at 4 °C with gentle rocking. The next day, the beads were washed three times with PBS. The pellet was then boiled in 2× SDS sample buffer, the supernatant was electrophoresed on a 7.5% SDS-polyacrylamide gel, and proteins were transferred to nitrocellulose. The membrane was then probed with a rabbit anti-MYC antiserum (1:1000, Cell Signaling Technology, #71D10), followed by anti-rabbit horseradish peroxidase-linked secondary antiserum (1: 4000, Cell Signaling Technology, #2368). Complexes were detected with SuperSignal West Dura Extended Duration substrate and an image was obtained on a Sapphire Biomolecular Imager, as described above. The membrane was stripped and probed with mouse anti-FLAG antiserum (1:1000, Cell Signaling, #8164) and goat anti-mouse horseradish peroxidase secondary antibody (1:4000, Cell Signaling, #7076. The filter was then imaged as described above.

### Indirect immunofluorescence

For immunofluorescence of transfected DF-1 cells, the media was removed, and cells were washed three times with room temperature PBS. Cells on coverslips were fixed in 100% methanol (pre-chilled at –20 °C) for 10 min. Coverslips were allowed to dry at room temperature for about 10–20 min on a paper towel. When dry, fixed cells on coverslips were blocked with PBS containing 3% calf serum (CaS; Sigma, #C8056) for 30 min at room temperature. Cells were then incubated with dilute primary antiserum (rabbit anti-FLAG, 1:80, Cell Signaling, #2368, or mouse anti-Myc, 1:100, Santa Cruz Biotechnology, #9E10) for 1 h at 37 °C. Coverslips were then washed 4× with PBS/3%CaS. Coverslips were then incubated with secondary antibody diluted 1:100 (goat anti-rabbit conjugated to Alexa Fluor-488, Invitrogen, #A32731, or goat anti-mouse conjugated to Alexa Fluor-555, Invitrogen, #A21422) for 1 h at 37 °C. Coverslips were then washed four times with PBS, and incubated with Hoechst 33342 (1:4000, Invitrogen, #H3570) for 15–20 min at room temperature. Coverslips were washed three times with PBS and then mounted on a glass slide with 25–40 μl of mounting medium (Slow Fade, Invitrogen, #S36937). Slides were then stored in the dark at either 4 °C (short-term) or –20 °C (long-term). Cells were imaged on a Nikon C2 Si confocal microscope at 405 nm (Hoechst), 488 nm (Alex Fluor-488), and 561 nm (Alexa Fluor-555). Images were taken with a Ti-E Spectral imaging system.

Indirect immunofluorescence of transfected *Capsaspora* cells was carried out essentially as described above for DF-1 cells, except *Capsaspora* cells were fixed with 4% paraformaldehyde for 20 min (instead of methanol). Cells were then blocked with PBS/3% CaS for 30 min. As described for DF-1 cells, *Capsaspora* cells were then incubated sequentially with anti-FLAG primary antiserum, Alexa Fluor-488-conjugated goat anti-rabbit secondary antiserum, and Hoechst 33342 stain before mounting with Slow-Fade. Like the DF-1 cells, slides were stored in the dark at either 4 °C (short-term) or –20 °C (long-term). Cells were imaged on a Nikon C2 Si confocal microscope at 405 nm (Hoescht) and 488 nm (Alexa Fluor-488). Images were taken with a Ti-E Spectral imaging system.

### Protein-binding microarray experiments and analysis

PBM experiments were carried out as described in refs. ^24,39^. Custom-made NF-κB oligonucleotide arrays (Agilent Technologies, AMADID 045485), which were based on a previously published 10-bp κB-site microarray used for human and mouse NF-κB proteins^27^. Proteins used for PBM analysis were GST-tagged expressed in BL21 bacterial cells, which were mechanically lysed using a French press in the presence of protease inhibitors. GST-tagged proteins were purified with glutathione agarose (ThermoFisher). Probes on PBMs were made double-stranded by incubating the array with dNTP annealing mix at 85 °C for 10 min, 75 °C 10 min, 65 °C 10 min, then 60 °C for 90 min. The array was blocked at room temperature in PBS with 2% milk for 1 h followed by washing in 0.1% PBS, Tween-20 (5 min) and 0.01% PBS, Triton-X (2 min). Arrays were incubated for 1 h at room temperature with ~300 nM protein in protein binding buffer (6 mM HEPES [pH 7.8], 80 mM KCl, 0.5 mM EDTA, 0.5 mM EGTA, 6% glycerol, 35 ng/µl poly-dI-dC, 1% milk). Arrays were then washed with PBS containing 0.05% Tween-20 and then PBS containing 0.01% Triton X-100. An Alexa Fluor 488-conjugated anti-GST antibody (1:40, Invitrogen) was used to detect bound proteins. After washing, arrays were scanned on a GenePix 4400 A Scanner (Molecular Devices) and fluorescence was quantified with GenePix Pro 7.2 (Molecular Devices). PBM probe fluorescence values were spatially averaged and normalized using MicroArray LINEar Regression^40^ as described^41^. Replicate experiments were combined using quantile normalization of probe fluorescence values using the *normalize.quantiles* method in the ‘R’ statistical package (www.r-project.org). For each unique DNA binding sequence, median fluorescence values were determined over eight replicate probe measurements. Log median fluorescence values (i.e., log(F)) were transformed into a ‘z-score’ using the mean (μ) and variance (σ) of the log median fluorescence values for the 1195 random background DNA sequences using the following equation:

3 $${{{{{\rm{z}}}}}}=(\log ({{{{{\rm{F}}}}}})-\mu )/ \left. \sigma\right)$$

To generate the scatter plots, *Co*-NF-κB sequence binding z-score values were compared to *Nv*-NF-κB, *Hu*-p52, *Hu*-RelA, and *Hu*-cRel, and plotted. The best fit line and R^2^ value were calculated by excluding z-scores lower than 2 for each comparison.

### RNA-sequencing analysis and annotation

RNA-seq data from each life stage of *Capsaspora* was obtained from Sebé-Pedrós et al.^16^. To identify potential target genes of NF-κB, we sorted all RNA-seq data to identify genes that were differentially expressed in the same manner as NF-κB for each life stage (lowest expression in aggregative, medium expression in filopodic, highest expression in cystic). We discarded genes that were not expressed in a given life stage (i.e., had an RPKM of 0). Of the 8674 total genes, 1348 genes were differentially expressed in a manner similar to *Co*-NF-κB. Of these 1348 genes, 389 genes had annotated homologs, and 305 had human homologs (Supplementary Data 2). We then performed GO analysis (http://pantherdb.org) by entering the UniProt ID of each gene and selecting *Homo sapiens*. We then created and analyzed the Biological Processes that were present in this gene list. To identify genes with potential upstream NF-κB binding sites among these 1348 genes, we extracted the 500 base pair sequence upstream of each gene. We then imported these 1348 upstream regions into MEME-FIMO and scanned the sequences using the PBM-generated*Co*-NF-κB motif (Supplementary Data 3).

### Statistics and reproducibility

Reporter gene assays in tissue culture cells (Figs. 2e and 6e) and yeast (Figs. 2f and 6f) were performed with multiple independent samples as described in Methods and figure legends, and are reported with standard error (as determined in Eq. 1). The sample sizes were as follows: Fig. 2e, *n* = 3 (with triplicate samples); Fig. 6e, *n* = 3 (with triplicate samples); Fig. 2f, *n* = 34, except *Co*-NF-κB, *n* = 8); and Fig. 6f, *n* = 8. For PBM analysis, the median fluorescence values for each unique DNA binding sequence were determined over eight replicate probe measurements. Log median fluorescence values were transformed into a ‘z-score’ using the mean (μ) and variance (σ) of the log median fluorescence values for the 1195 random background DNA sequences. The best fit line and R^2^ values for the scatter plots were calculated by excluding z-scores lower than 2 for each comparison. Other experiments, e.g., EMSAs, Western blots, were performed at least two times and had similar results to the ones reported here. Moreover, such experiments have internal controls for molecular weight markers and loading accuracy.

### Reporting summary

Further information on research design is available in the Nature Research Reporting Summary linked to this article.

## Supplementary information

Supplementary Information

Description of Additional Supplementary Files

Supplementary Data 1

Supplementary Data 2

Supplementary Data 3

Supplementary Data 4

Supplementary Data 5

Reporting Summary

## Data Availability

All data generated and analyzed during this study are included in this published article (and its Supplementary information files). The raw images for Western blots and EMSAs can be found in Supplementary Figs. 10–14. The raw data for reporter assays can be found in Supplementary Data 5. *Capsaspora*RNA-Seq data for different life stages were taken ref. ^16^, 10.6084/m9.figshare.4585447.v2. *Capsaspora* proteomic and phosophoproteomic data from were taken from ref. ^17^, Accession number PRIDE: PXD004567. Data for choanoflagellate NF-κB proteins were taken from ref. ^5^, Accession at NCBI SRA under BioProject PRJNA419411. Additional data relating to the study are available from the corresponding author upon reasonable request.
