# Supplementary material for: Comparison of NF-κB from the protists Capsaspora owczarzaki and Acanthoeca spectabilis reveals extensive evolutionary diversification of this transcription factor
Source: Commun Biol. 2021 Dec 16;4:1404. doi: 10.1038/s42003-021-02924-2 (PMC8677719; doi:10.1038/s42003-021-02924-2)
Supplement: Supplementary file 1 — Supplementary Information [file 42003_2021_2924_MOESM1_ESM.pdf]

Supplementary Material for

Comparison of NF-κB from the protists *Capsaspora owczarzaki* and *Acanthoeca spectabilis* reveals extensive evolutionary diversification of this transcription factor

Leah M. Williams, Sainetra Sridhar, Jason Samaroo, Jada Peart, Ebubechi K. Adindu, Anvitha Addanki, Christopher J. DiRusso, BB522 Molecular Biology Laboratory, Pablo J. Aguirre Carrión, Nahomie Rodriguez-Sastre, Trevor Siggers, Thomas D. Gilmore,*

Department of Biology, Boston University, Boston, MA 02215, USA

*Dr. Thomas D. Gilmore, Biology Department, Boston University, 5 Cummington Mall, Boston, MA 02215, USA; 617-353-5444 (phone); 617-353-6340 (fax); email: gilmore@bu.edu

This file contains Supplementary Tables, Supplementary Figures, and Supplementary References

Supplementary Tables

Supplementary Table 1. Predicted molecular weights of protist NF-κBs and mutant proteins.

Protein Name	# of amino acids (including FLAG)	Predicted mol. weight (kDa)
FLAG-Nv-NF-κB	447	49.4
FLAG-Co-NF-κB	1231	129.9
FLAG-Co-RHD	589	62.5
FLAG-Co-Cterm	689	71.9
FLAG-As-NF-κB1	416	44.7
FLAG-As-NF-κB2	508	55.9
FLAG-As-NF-κB3	418	46.7

Supplementary Table 2. Quantification of subcellular localization of NF-κB-positive cells in transfected DF-1 cells analyzed by indirect immunofluorescence.

Transfected Plasmid	Nucleus	Cytoplasm	Cytoplasm/Nucleus
FLAG-*Nv*-NF-κB	79/79 (100%)	0/79	0/79
FLAG-*Co*-NF-κB	1/702 (0.1%)	701/702 (99.9%)	0/702
FLAG-*Co*-RHD	1874/2114 (88.7%)	19/2114 (0.9%)	221/2114 (10.5%)
FLAG-*Co*-Cterm	3/570 (0.5%)	536/570 (94%)	31/570 (5.4%)

Values represent the number of cells with the indicated subcellular localization over the total cells counted for the given transfected plasmid (value as a percentage).

Supplementary Table 3. ***Capsaspora*** **gene homologs that are annotated within the genome that are in the Developmental and Immune system process GO categories. The genes highlighted in red are in both lists.**

**Developmental Process Genes in Capsaspora (47 genes)**	**Immune System Process Genes in Capsaspora (26 genes)**
actin-like protein 2	ankyrin repeat domain-containing protein
adenylyl cyclase-associated protein	ADP-ribosyl cyclase
ankyrin repeat domain-containing protein	ATP-binding cassette
Coronin	ATP-dependent RNA helicase
cyclin-dependent kinase	cactin
cytoplasmic FMR1-interacting protein 1	cathepsin L2
E3 ubiquitin-protein ligase NEDD4	heat shock protein
ephrin type-B receptor 3	hydrolase
flightless-1	integral membrane protein
G protein-coupled receptor	mitogen-activated protein kinase kinase kinase (SLK)
GTP-binding protein	peroxiredoxin-2
GTPase	phospholipase A2
guanine nucleotide exchange factor	programmed cell death protein
integral membrane protein	protein kinase C
kinesin-1	protein tyrosine kinase
leucine-rich repeat-containing protein	ring finger protein
microtubule-associated protein	RNA helicase
mitogen-activated protein kinase kinase kinase (SLK)	RNA-binding protein
myosin-VI	SH3 domain-containing protein
myosin-VIIa	transcriptional regulator
Myotubularin	tyrosine-protein kinase CSK
nucleosome assembly protein	tyrosine-protein kinase ITK/TSK
nucleotide pyrophosphatase/phosphodiesterase	tyrosine-protein kinase Src42A
Paxillin	tyrosine-protein kinase Srms
phosphoenolpyruvate carboxykinase	tyrosine-protein kinase SYK
Phosphoglucomutase	zinc finger protein
protein phosphatase 2	
rho GTPase-activating protein	
serine/threonine-protein kinase 4	

SPRY domain-containing protein	
T-box transcription factor TBX4	
Thrombospondin	
transcriptional regulator	
tubulin-beta chain	
twinfilin-1	
Tyrosine-protein kinase Fyn (SLK)	
tyrosine-protein kinase ITK/TSK	
tyrosine-protein kinase Src42A	
tyrosine-protein kinase Srms	
tyrosine-protein kinase SYK	
tyrosine-protein kinase transmembrane receptor Ror	
ubiquitin-protein ligase	
vasodilator-stimulated phosphoprotein	
vesicle-associated membrane protein	
Villin	
WD-repeat protein 50	
NT-3 growth factor receptor NTRK3	

Supplementary Table 4. Primers used in this study.

Primers used for subcloning (restriction enzyme sites used for subcloning are underlined where necessary)

Primer Name	Primer Sequence
F-Gib-RHD-1	5’-CTGGTGGGGTCGTGAAACGGCGTTAAGACTCAGGG-3’
R-Gib-RHD-1	5’-GGTACCATGGACTACAAGGACG -3'
F-Gib-Cterm-2	5’- CCGTTTTCACGACCCCACCAGAATC -3’
R-Gib-Cterm-2	5’- CTAGCATTTAGGTGACACTATAGAATAGGG -3'
F-Gib-FLAG-3	5’-CTATTCTATAGTGTCACCTAAATGCTAGAGC-3’
R-Gib-FLAG-3	5’-CCTTGTAGTCCATGGTACCAAGC-3'
F-FLAG-Co-NF-κB	5’-CGCGCGAATTCAGACCTTTCTGAACTTTCCGGATGGG-3’
R-FLAG-Co-RHD	5’-GCGCGCTCGAGTTACCCGCCTTTACTTGAAGAACTCCC-3'
F-FLAG-Co-ANK	5'-CGCGCGAATTCAGATGGGTCCGACGGTGGAAATG-3'
R-FLAG-Co-NF-κB	5'-GCGCGCTCGAGTTAATCAACGGAGTAAAGGGCATGGG-3'
F-GBT9-Co-NF-κB	5'-CGCGCGAATTCGACCTTTCTGAACTTTCCGGATGGG-3'
R-GBT9-Co-NF-κB	5'-GCGCGCTCGAGTTAATCAACGGAGTAAAGGGCATGGGT-3'
R-GBT9-Co-RHD	5'-GCGCGCTCGAGTTACCCGCCTTTACTTGAAGAACTCCC-3'
F-GBT9-Co-Cterm	5'-CGCGCGAATTCGATGGGTCCGACGGTGGAAATG-3'
F-GBT9-As-NF-κB1-EcoRI	5'-CGCGCGAATTCGAAGCCAGATGGCGGCGGGCCCCTGGCCTGG-3'
R-GBT9-As-NF-κB1-SalI	5'-CGCGCGTCGACTCACATCAGGGCAGGGCCAGGGGCGGCATCACTAGC-3'
F-GBT9-As-NF-κB2-EcoRI	5'-CGCGCGAATTCGAGCCCTGGCGAACACGCCAATACTCAC-3'
R-GBT9-As-NF-κB2-SalI	5'-CGCGCGTCGACTTAAACGGAAGTCTCGAAGGACCCGTATACCCTCC-3'
F-GBT9-As-NF-κB3-EcoRI	5'-CGCGCGAATTCCCAAATGATTTCGAGGCTGTTCTGACGTCTGGGG-3'
R-GBT9-As-NF-κB3-SalI	5'-CGCGCGTCGACTCATTCGGGTGGTGGTCTGCGCCTTC-3'
F-MYC-As-NF-κB1-EcoRI	5'-GATGACAAGTTAACACGTGCTGC-3'
R-MYC-As-NF-κB1-EcoRI	5'-CGCGCGAATTCTCACATCAGGGCAGGGC-3'
F-MYC-As-NF-κB2-EcoRI	5'-GAATTCTGAGCCCTGGCGAACAC-3'
R-MYC-As-NF-κB2-XbaI	5'-TCTAGATTAAACGGAAGTCTCGAAGGAC-3'
F-MYC-As-NF-κB3-EcoRI	5'-GAATTCTCCAAATGATTTCGAGGCTGTTC-3'
R-MYC-As-NF-κB3-XbaI	5'-TCTAGATCATTCGGGTGGTGGTC-3'
MYC-Co-NF-κB-XhoI	5'-GCGCGTCTAGATTAATCAACGGAGTAAAGGGCATGGG-3'

Primers for EMSA. The κB site is underlined. Primers were annealed before use in EMSA.

Consensus NF-κB binding site	5’- TCGAGAGGTCGGGGAATTCCCCCCCCG -3’
	5’- TCGACGGGGGGGGAATTCCCCGACCTC -3’

Supplementary Table 5. Plasmids used in this study

Expression vectors for use in *Capsaspora*, tissue culture, and yeast cells

Plasmid Name	Plasmid Description
pcDNA-FLAG	pcDNA with a 5’ FLAG Tag (1).
pcDNA-FLAG-Nv-NF-κB	Ref. 1
pcDNA-FLAG-Co-NF-κB	EcoRI-XhoI fragment containing amino acids 2-1224 of Co-NF-κB was excised from pUC57-Co-NF-κB (GenScript) and subcloned into EcoRI-XhoI-digested pcDNA-FLAG.
pcDNA-FLAG-Co-NF-κB-ALA	Gibson primers were used to amplify off pcDNA FLAG-Co-NF-κB and pcDNA FLAG Aiptasia NF-κB-AAA (2) to create three fragments which were assembled using the Gibson Assembly Cloning Kit (New England BioLabs). Primers F-Gib-RHD-1 and R-Gib-RHD-1 were used to create a fragment that contained the FLAG-tag and Co-NF-κB aa 2-889; Primers F-Gib-Cterm-2 and R-Gib-Cterm-2 were used to create a fragment that contained the C-terminus of pcDNA FLAG *Aiptasia* NF-κB-SSS; Primers F-Gib-FLAG-3 and R-Gib-FLAG-3 were used to create the pcDNA FLAG backbone.
pcDNA-FLAG-Co-NF-κB-SER	Gibson primers were used to amplify off pcDNA FLAG-Co-NF-κB and pcDNA FLAG Aiptasia NF-κB (2) to create three fragments which were assembled using the Gibson Assembly Cloning Kit (New England BioLabs). Primers F-Gib-RHD-1 and R-Gib-RHD-1 were used to create a fragment that contained the FLAG-tag and Co-NF-κB aa 2-889; Primers F-Gib-Cterm-2 and R-Gib-Cterm-2 were used to create a fragment that contained the C-terminus of pcDNA FLAG *Aiptasia* NF-κB; Primers F-Gib-FLAG-3 and R-Gib-FLAG-3 were used to create the pcDNA FLAG backbone.
pcDNA-FLAG-Co-RHD	EcoRI-XhoI digested Co-RHD PCR product containing aa 2-582 was subcloned into EcoRI-XhoI digested pcDNA-FLAG. Primers: F-FLAG-Co-NF-κB and R-FLAG-Co-RHD. PCR-amplified from pcDNA-FLAG-Co-NF-κB.
pcDNA-FLAG-Co-Cterm	EcoRI-XhoI digested Co-Cterm PCR product containing aa 542-1224 was subcloned into EcoRI-XhoI digested pcDNA-FLAG. Primers: F-FLAG-Co-ANK and R-FLAG-Co-NF-κB. PCR- amplified from pcDNA-FLAG-Co-NF-κB.
pUC57-Co-NF-κB	pUC57-Simple with Co-NF-κB cDNA codon-optimized for expression in human cells. Has a 5’ EcoRI site and 3’ XhoI site for excision. Synthesized by GenScript.
pcDNA-FLAG-Ap-IKK	Ref. 2

pcDNA-FLAG-IKKβ-SS-EE	Ref. 2
HA-NIK	Ref. 2
HA-IKKβ-SS-EE	Ref. 2
GBT9	Ref. 1
GB-Nv-NF-κB	Ref. 1
GB-Co-NF-κB	EcoRI-XhoI digested Co-NF-κB PCR product containing codons 2-1224 was subcloned into EcoRI-XhoI digested pGBT9 vector. Primers F-GBT9-Co-NF-κB and R-GBT9-Co-NF-κB were used to PCR-amplify the fragment from pcDNA-FLAG-Co-NF-κB.
GB-Co-RHD	EcoRI-XhoI digested Co-RHD PCR product containing codons 2-582 was subcloned into EcoRI-XhoI digested pGBT9 vector. Primers F-GBT9-Co-NF-κB and R-GBT9-Co-RHD were used to PCR-amplify the fragment from pcDNA-FLAG-Co-NF-κB.
GB-Co-Cterm	EcoRI-XhoI digested Co-Cterm PCR product containing codons 542-1224 was subcloned into EcoRI-XhoI digested pBGT9 vector. Primers F-GBT9-Co-Cterm and R-GBT9-Aq-NF-κB were used to PCR amplify the fragment from pcDNA-FLAG-Co-NF-κB.
pUC57-As-NF-κB1	pUC57-Simple with As-NF-κB1 cDNA codon-optimized for expression in human cells. Has a 5' and 3' BamHI sites for excision. Synthesized by GenScript.
pUC57-As-NF-κB2	pUC57-Simple with As-NF-κB2 cDNA codon-optimized for expression in human cells. Has a 5' and 3' EcoRI sites for excision. Synthesized by GenScript.
pUC57-As-NF-κB3	pUC57-Simple with As-NF-κB3 cDNA codon-optimized for expression in human cells. Has a 5' and 3' EcoRI sites for excision. Synthesized by GenScript.
pcDNA FLAG-As-NF-κB1	BamHI-BamHI fragment containing amino acids 2-409 of As-NF-κB1 was excised from pUC57-As-NF-κB1 and subcloned into BamHI-digested pcDNA-FLAG.
pcDNA FLAG-As-NF-κB2	EcoRI-EcoRI fragment containing amino acids 2-502 of As-NF-κB2 was excised from pUC57-As-NF-κB2 and subcloned into EcoRI-digested pcDNA-FLAG.
pcDNA FLAG-As-NF-κB3	EcoRI-EcoRI fragment containing amino acids 2-412 of Co-NF-κB was excised from pUC57-As-NF-κB3 and subcloned into EcoRI-digested pcDNA-FLAG.

GB-As-NF-κB1	EcoRI-SalI digested As-NF-κB1 PCR product containing codons 2-409 was subcloned into EcoRI-SalI digested pGBT9 vector. Primers F-GBT9-As-NF-κB1-EcoRI and R-GBT9-As-NF-κB1-SalI were used to PCR amplify the fragment from pcDNA-FLAG-As-NF-κB1.
GB-As-NF-κB2	EcoRI-SalI digested As-NF-κB2 PCR product containing codons 2-502 was subcloned into EcoRI-SalI digested pGBT9 vector. Primers F-GBT9-As-NF-κB2-EcoRI and R-GBT9-As-NF-κB2-SalI were used to PCR amplify the fragment from pcDNA-FLAG-As-NF-κB2.
GB-As-NF-κB3	EcoRI-SalI digested As-NF-κB3 PCR product containing codons 2-412 was subcloned into EcoRI-SalI digested pGBT9 vector. Primers F-GBT9-As-NF-κB3-EcoRI and R-GBT9-As-NF-κB3-SalI were used to PCR amplify the fragment from pcDNA-FLAG-As-NF-κB3.
pcDNA MYC vector	Ref. 1
MYC-As-NF-κB1	EcoRI-XbaI fragment containing amino acids 2-409 of As-NF-κB1 was PCR-amplified from pcDNA FLAG-As-NF-κB1 using primers F-MYC-As-NF-κB1-EcoRI and R-MYC-As-NF-κB1-EcoRI. Fragment was then subcloned into EcoRI-XbaI digested pcDNA-MYC vector.
MYC-As-NF-κB2	EcoRI-XbaI fragment containing amino acids 2-502 of As-NF-κB2 was PCR-amplified from pcDNA FLAG-As-NF-κB2 using primers F-MYC-As-NF-κB2-EcoRI and R-MYC-As-NF-κB2-XbaI. Fragment was then subcloned into EcoRI-XbaI digested pcDNA-MYC vector.
MYC-As-NF-κB3	EcoRI-XbaI fragment containing amino acids 2-412 of As-NF-κB3 was PCR-amplified from pcDNA FLAG-As-NF-κB3. Fragment was then subcloned into EcoRI-XbaI digested pcDNA-MYC vector.
MYC-Co-Cterm	EcoRI-XbaI fragment containing amino acids 542-1224 of Co-NF-κB was PCR-amplified from pcDNA FLAG-Co-NF-κB. Fragment was then subcloned into EcoRI-XbaI digested pcDNA-MYC vector.
3X-κB-luc	Ref 1

Supplementary Figures

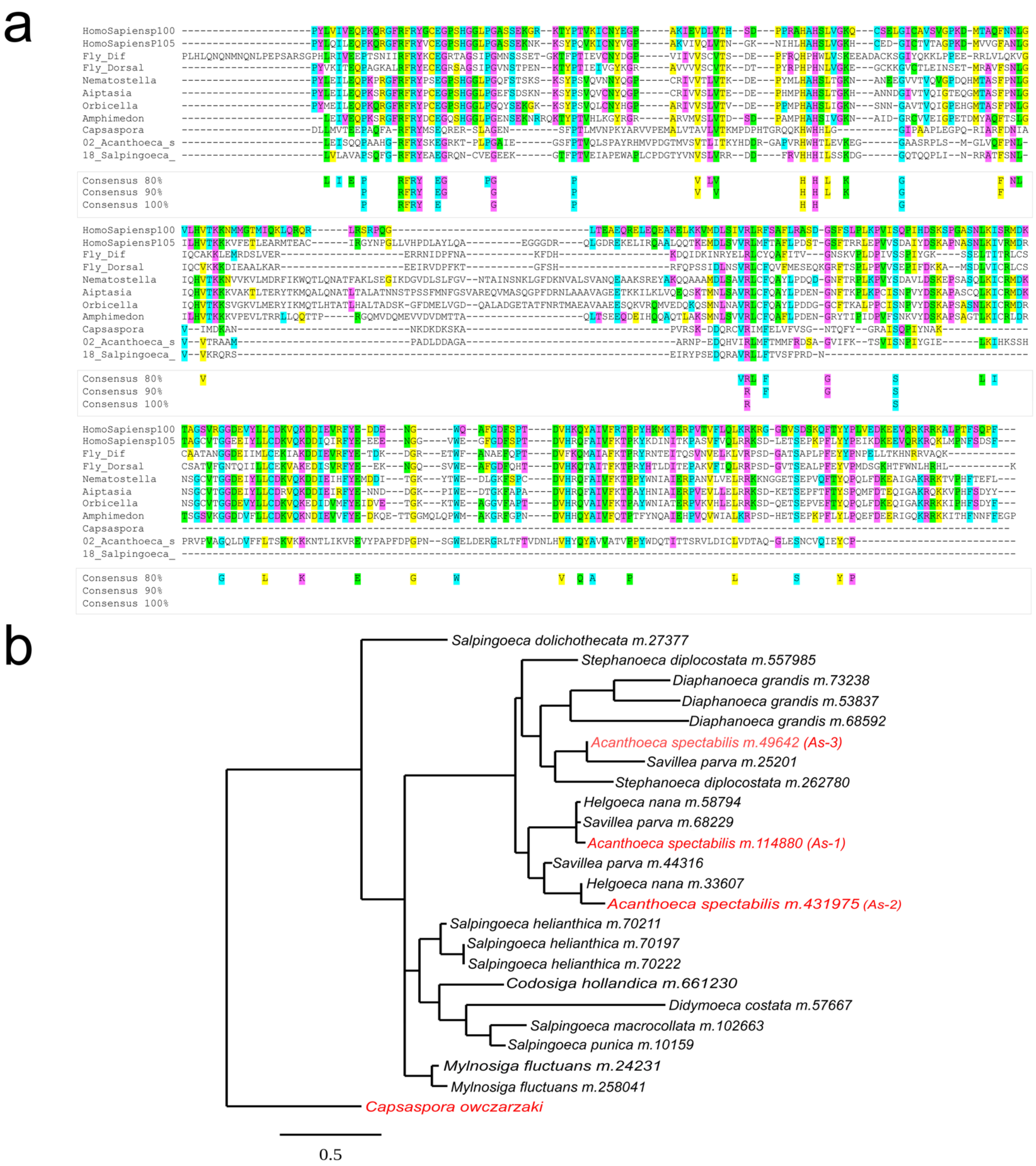

Supplementary Fig. 1. (a) RHD sequence alignments of NF-κBs from recently derived organisms and three protists (*Capsaspora*, *Acanthoeca spectabilis As*-NF-κB1 [protein ID m.114880], and *Salpingoeca helianthica* [protein ID m.70197]). The percent identities of given conserved residues are highlighted underneath the sequences. (b) A phylogenetic estimation using maximum likelihood of choanoflagellate and *Capsaspora* RHDs, with protein IDs (taken from Dataset 4 of ref. 3) indicated after the name. Scale bar = number of substitutions per site. The NF-κB proteins used in these studies are highlighted in red. See ref. 4 for a more extensive version of these analyses.

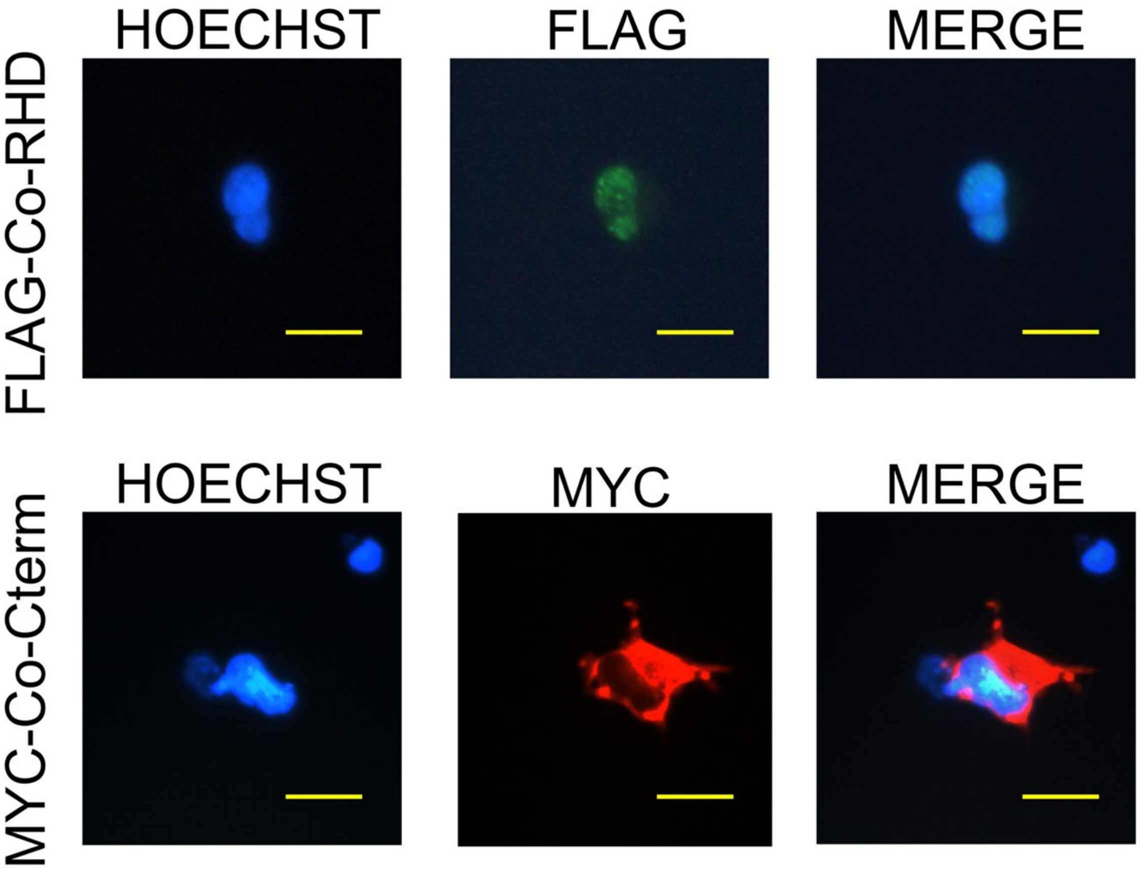

Supplementary Fig. 2. DF-1 chicken fibroblast cells were transfected with empty vector and either FLAG-*Co*-RHD (top row) or MYC-*Co*-Cterm (bottom row). As indicated, cells were then stained with either anti-FLAG (green) or anti-MYC (red) primary antiserum, and Hoechst (blue) for staining nuclei. Yellow scale bar is is 10 µm.

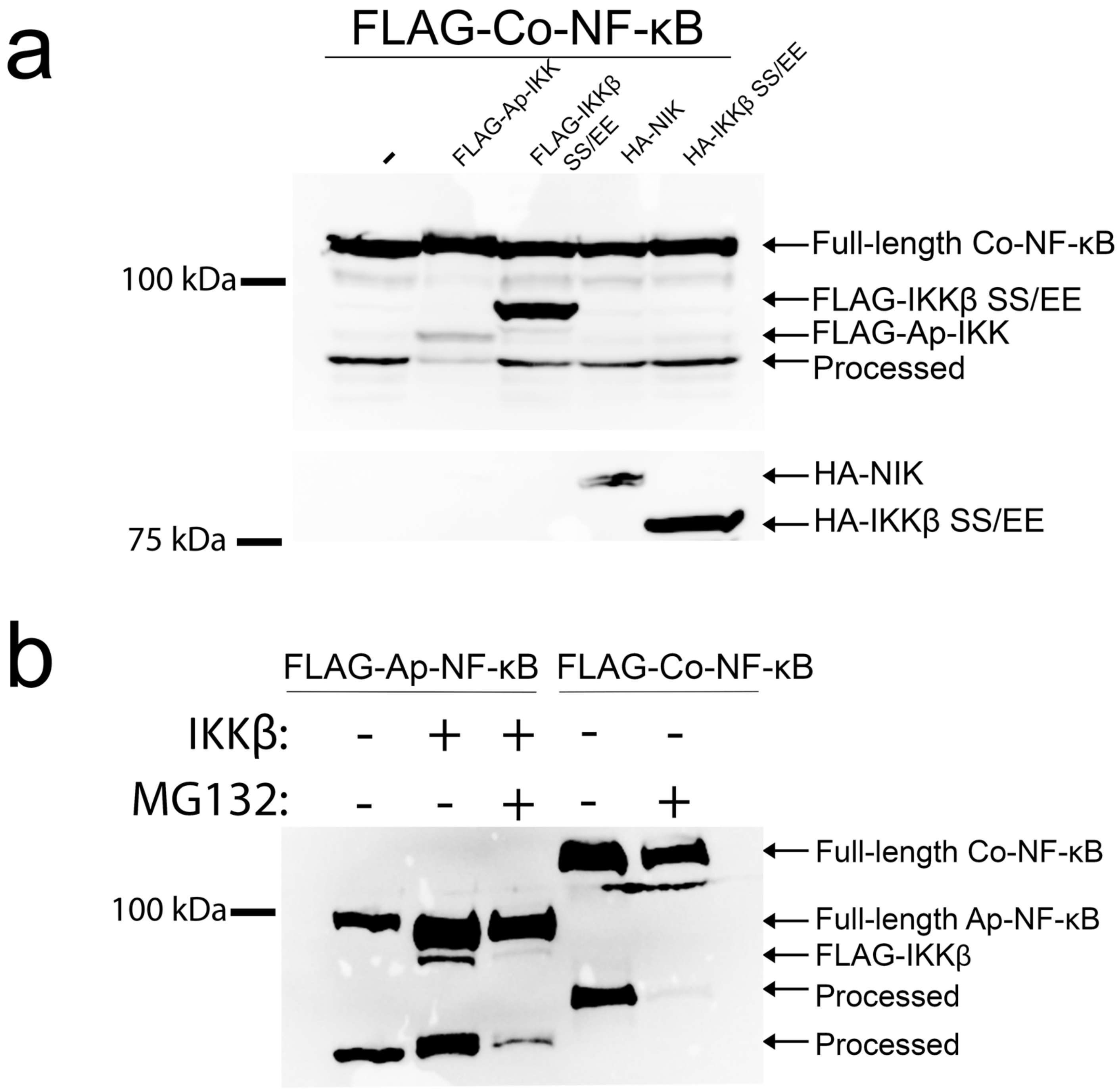

Supplementary Fig. 3. (a) Co-transfection with various IKK family kinases does not induce processing of FLAG-*Co*-NF-κB in HEK 293T cells. Arrows indicate the various FLAG- or HA-tagged kinases used in these assays. Full-length *Co*-NF-κB and processed *Co*-NF-κB are also indicated. (b) HEK 293T cells were transfected with FLAG-*Ap*-NF-κB or FLAG-*Co*-NF-κB and either a vector control or a FLAG-human IKKβ expression

vector. Where indicated MG132 (40 uM) was added for 16 h prior to lysis. Cells were then lysed and anti-FLAG Western blotting was performed as in Fig. 3. Raw images are shown in Supplementary Figure 14.

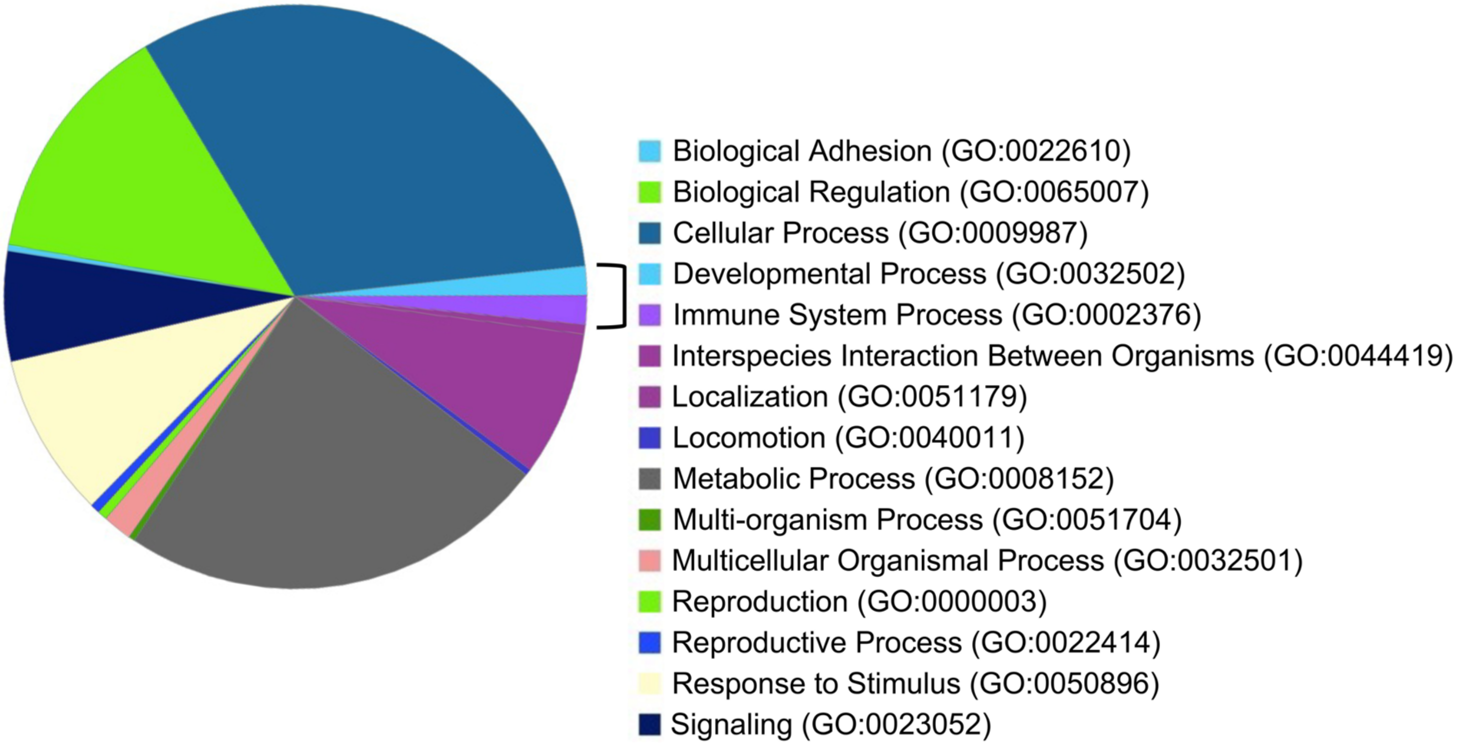

Supplementary Fig. 4. GO analysis was performed on the 305 annotated *Capsaspora* gene homologs that show the same expression pattern as *Co*-NF-κB mRNA across the three *Capsaspora* life stages. The top 15 GO terms with GO term numbers (right) and their relative abundances (left pie chart) are shown.

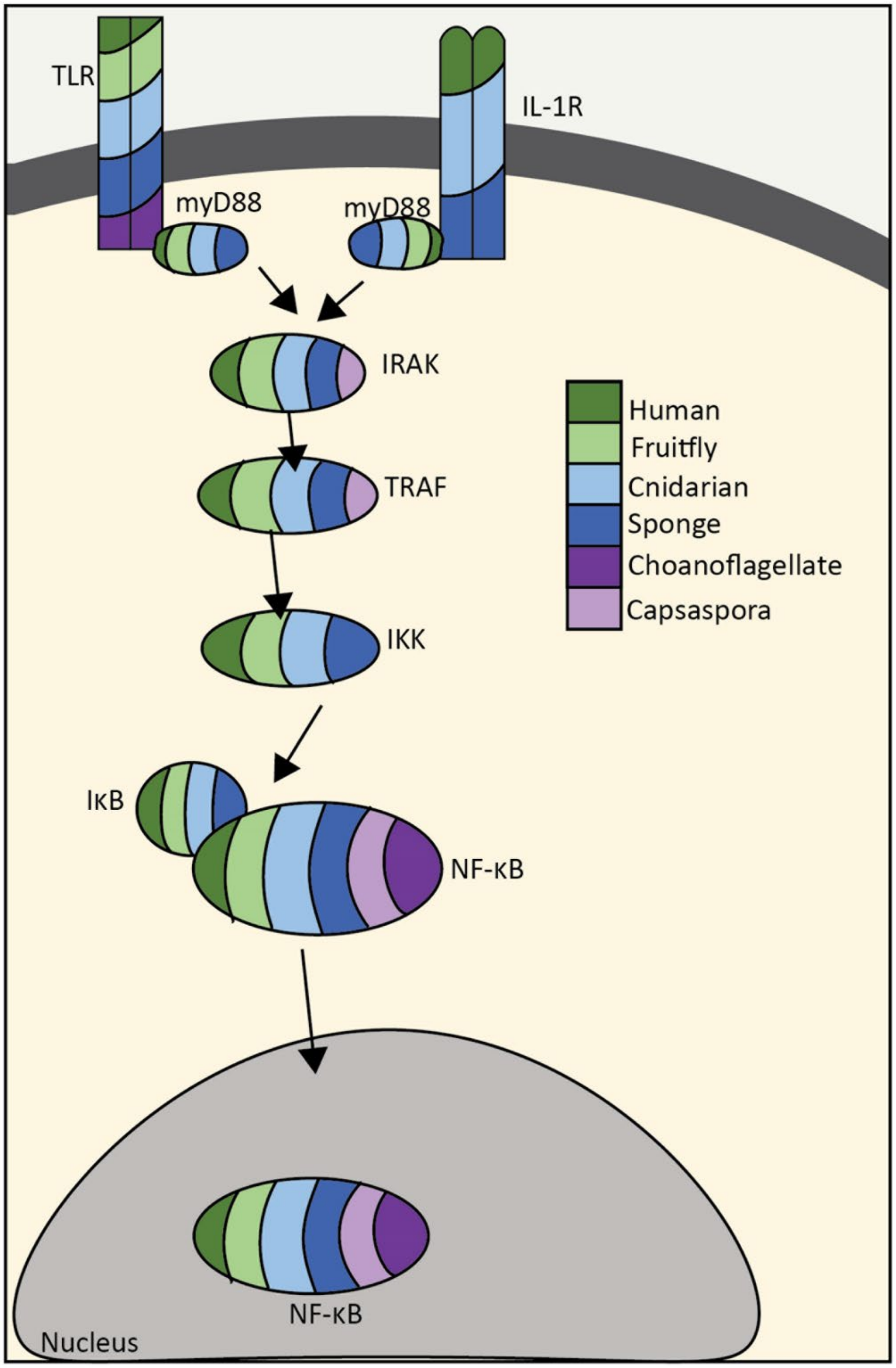

Supplementary Fig. 5. Schematic of the proteins found in the simplified TLR pathway of various organisms. For each protein, the presence of a homolog is indicated according to the color legend on the right for the indicated organisms.

atg gac ctt tct gaa ctt tcc gga tgg gac ccg aat ttg tcc ttg cag gag cac act gca
M D L S E L S G W D P N L S L Q E H T A
aat ctg ctc gct atg gat gat tca aca atg gca gct atg ata ctg cat tct gat gga ctg
N L L A M D D S T M A A M I L H S D G L
tca ttg ttc aga gat atg ggt ctg tac aat tcc gtc tct acg agc ctg gat att agc ggt
S L F R D M G L Y N S V S T S L D I S G
att cca aaa ttt ccg cca ccg cca cag cag ccg caa ctt gca caa gcg ccg cct cgg aga
I P K F P P P P Q Q P Q L A Q A P P R R
ggt cac aac caa tcc agc agt agc gat agc cat agc aca cca tca ccc ggt tcc gtg ctc
G H N Q S S S S D S H S T P S P G S V L
ttc tcc ccg agc ccg gct tct caa gat atg agc ctc cag agt ccg gag ctg ggc ctc gtt
F S P S P A S Q D M S L Q S P E L G L V
ggg ctt act ggc aac agt agg ctg gcg tca acg tca gag gcc gat gat att ctg ctt gcg
G L T G N S R L A S T S E A D D I L L A
aat ata ctg ggg cac cca gta cgg tca gtt agt gca aat aca tca atg gtt ggt ctt ccg
N I L G H P V R S V S A N T S M V G L P
gat gac ctg gga ttc tcc ccc acg acc gga atg gat ctc aca atc acg gcg acg agt cca
D D L G F S P T T G M D L T I T A T S P
gcg acg gca gac tca tcc gcg tca gct aca gcc ttc cct gcg gcg tcc ccg ata gct tca
A T A D S S A S A T A F P A A S P I A S
cct agc gtc tcc acc agc tcc ggg cct gta act gtt ggc gga acc agc gcg gcc ttg gcc
P S V S T S S G P V T V G G T S A A L A
gca ttg act ccg gac aga att aat agg ctt ctt agc gca gcc gag gcg gca gcc gaa ggg
A L T P D R I N R L L S A A E A A A E G
gcg gcc acc ctc gtt gag gac ctt ctt atg gtc acg gag gag ccc gcg cag ttc gcc cga
A A T L V E D L L M V T E E P A Q F A R
ttc cgc tac atg agc gag cag cga gag aga tct ttg gcc ggc gag aac tcc ttc ccc acg
F R Y M S E Q R E R S L A G E N S F P T
ctt atg gta aac cct aag tat gcc aga gtg gtc ccc gag atg gcc ctc gta act gct gtc
L M V N P K Y A R V V P E M A L V T A V
ctg gtc aca aaa atg ccg gat ccc cac act ggg agg cag cag aag cat tgg cat cac ctt
L V T K M P D P H T G R Q Q K H W H H L
ggg ggc ata cct gcg gct cct ctg gaa ggg cct caa cgc ata gca agg ttc gac aac att
G G I P A A P L E G P Q R I A R F D N I
gct gta ata atg gac aag gcg aac aat aag gat aag gac aaa tca aag gca ccc gta aga
A V I M D K A N N K D K D K S K A P V R
tct aaa gac gat cag cga tgt gtc agg ata atg ttt gaa ctt gtc ttt gtg tca ggt aat
S K D D Q R C V R I M F E L V F V S G N
aca cag ttc tat ggc agg gca atc agc caa cca atc tac aac gcc aaa ttg gcc atc aca
T Q F Y G R A I S Q P I Y N A K L A I T
aag att agt cat agc agc ggg ccc gtg acc ggt ggt aac gag gtt atc atg ttg tgc agt
K I S H S S G P V T G G N E V I M L C S
aag atc aga aaa ggc gtg acc ggg gta cgg atg acc gat cca acc caa tgg tca gtc cag
K I R K G V T G V R M T D P T Q W S V Q
gca cct agc ggc tct gcg tgg gag ttg aat cca caa acg ctg aag gcc gac tgc aat gta
A P S G S A W E L N P Q T L K A D C N V
ccg ggc gct aat ctt ttt ttc cat cat caa tat gca gtc gtc ctg acg ctg ccg ccc tac
P G A N L F F H H Q Y A V V L T L P P Y
cac acg cag acc ata aca gcc cct gtc acc gtc agg atc tca atc ttg gac act gac gat
H T Q T I T A P V T V R I S I L D T D D
gag aca gag agt caa tac gtt gag tat acc tat ttg ccg gcc gaa gcg gcg gtg cgc aac
E T E S Q Y V E Y T Y L P A E A A V R N
gca gag ctg gcg gct cgg aaa cgg aga cgg gat gat tca atg aga gat ttt atg gac cgc
A E L A A R K R R R D D S M R D F M D R
ttt gat ggg tcc gac ggt gga aat ggc tct ggg agt ggc cga ggt aac aac gga gga cac
F D G S D G G N G S G S G R G N N G G H
gat ggc tct gac gct aac aat aac ggc cga ggc ggt ggc ggt ggg agt tct tca agt aaa
D G S D A N N N G R G G G G G S S S S K
ggc ggg gac gaa ccg ttt aat ttt aat tca ctc att cca atg cac caa cac aag ctt cat
G G D E P F N F N S L I P M H Q H K L H

```
caa ctg gcg ctc tcc acg gta agg gct gtg caa ggg ttc gcg gcc agt ggg gat gca cgg
 Q   L   A   L   S   T   V   R   A   V   Q   G   F   A   A   S   G   D   A   R
tat ctg ttg gcc ttg cat aga cag ttg ctt gca gct ccg aac gaa aac ggt gac tcc ccg
 Y   L   L   A   L   H   R   Q   L   L   A   A   P   N   E   N   G   D   S   P
ctg cac aca gca gta gca cag ggt aat ctc aga tct act atg gca ttg ctc cca ttg ctt
 L   H   T   A   V   A   Q   G   N   L   R   S   T   M   A   L   L   P   L   L
gcg gca gaa gat ctc caa agt gtg aac gac atg ggg gaa act gtc ttg cat agc gct gta
 A   A   E   D   L   Q   S   V   N   D   M   G   E   T   V   L   H   S   A   V
att gag aaa agg gca gcc ata gct cgg ctg ctt ctg gtc gct ggc gca gat ctt ggc cag
 I   E   K   R   A   A   I   A   R   L   L   L   V   A   G   A   D   L   G   Q
agt aat gca cgg aac ttt aat cga aac agc ctt cac tac ctt gcc cgg cat gga gat agg
 S   N   A   R   N   F   N   R   N   S   L   H   Y   L   A   R   H   G   D   R
gcg act gca atg gct gtg ttc ggc gtt ttt ggc agt gcg caa gcc cca cca gct aac acg
 A   T   A   M   A   V   F   G   V   F   G   S   A   Q   A   P   P   A   N   T
aac acg ccc gca cag gca ccg gca ggc gaa acg aag ccg aaa cca gct gac ttg aga ctg
 N   T   P   A   Q   A   P   A   G   E   T   K   P   K   P   A   D   L   R   L
ttg gca agg att cag gcg cag gct ata aaa gcg ctc ctg gcc tgt gaa ctc gaa act ggg
 L   A   R   I   Q   A   Q   A   I   K   A   L   L   A   C   E   L   E   T   G
gcc act ccc gcc cat ttg gcg ata cgg ggt ggc cac tgg cac gtc ttt gaa gcg tgt gct
 A   T   P   A   H   L   A   I   R   G   G   H   W   H   V   F   E   A   C   A
aag ttg gcg gca tca gca ccc ata ccc aaa gcc gct ggt tct ctt ctt tct atg gtc gct
 K   L   A   A   S   A   P   I   P   K   A   A   G   S   L   L   S   M   V   A
gaa aag agt agt gga cat agt ctg ttg cac tcc tgc gtc ctg gcc aat aat gag cag gct
 E   K   S   S   G   H   S   L   L   H   S   C   V   L   A   N   N   E   Q   A
gtt cga ctt ctt ata aat ctg ggg gcc tct ggc aac gct agg gac ttc ggg aag aac acg
 V   R   L   L   I   N   L   G   A   S   G   N   A   R   D   F   G   K   N   T
cca ctt cac ctg gcc gca cga caa gga cac atc ggc ata gcc gcg ctg ttg gta gag gca
 P   L   H   L   A   A   R   Q   G   H   I   G   I   A   A   L   L   V   E   A
ggg gct acc ctg agt ctt aac gcc gtt agc cag act ccc ctc gat gtt ttg act tca gag
 G   A   T   L   S   L   N   A   V   S   Q   T   P   L   D   V   L   T   S   E
ggt tcc gga ttg agc aga gat cag ctc cgg gca ttg gta gca gtc ctc cga ggc gag ctc
 G   S   G   L   S   R   D   Q   L   R   A   L   V   A   V   L   R   G   E   L
aaa tat gcc gac atg cgc ggc cgg cca acc ctc cga atg ccg act cac gcg gag ctg cat
 K   Y   A   D   M   R   G   R   P   T   L   R   M   P   T   H   A   E   L   H
agc acg gcg gcc gca ctc act tcc gcc tcc cca ggg gca gtc tca ctg gct gat ttc tac
 S   T   A   A   A   L   T   S   A   S   P   G   A   V   S   L   A   D   F   Y
gcg ggc aaa aag gcc tca agg agc ccc gcc cct ctc ggt gct tcc tca agt ctt ctt agt
 A   G   K   K   A   S   R   S   P   A   P   L   G   A   S   S   S   L   L   S
agt act gga gct tct gcg gct ggc gcc tct gcg ccc acg ata gcg gcg gtg cat gcc gcg
 S   T   G   A   S   A   A   G   A   S   A   P   T   I   A   A   V   H   A   A
agc gca aca ccg gta gag cgc acc agc atg aac aat gac gat gat tac gtt ctc ctt gag
 S   A   T   P   V   E   R   T   S   M   N   N   D   D   D   Y   V   L   L   E
aag gac gcg cct tac cct gtt gag cag cag cct cac ggg aag cgc aac aag cat tct cac
 K   D   A   P   Y   P   V   E   Q   Q   P   H   G   K   R   N   K   H   S   H
cac aga ttc acc cgg tcc agt cac ggc tca cag gat aaa gat gag ctt aaa aaa gat aag
 H   R   F   T   R   S   S   H   G   S   Q   D   K   D   E   L   K   K   D   K
gat gac ccg aaa aaa gaa aaa gag ccg aag gaa ctg tca aaa ttc aca ctg aaa gag gcg
 D   D   P   K   K   E   K   E   P   K   E   L   S   K   F   T   L   K   E   A
ttt gtg gac ggt acg aat ttt tgg gaa ctt act cgg aaa ttc gct ggg aag aag aaa atg
 F   V   D   G   T   N   F   W   E   L   T   R   K   F   A   G   K   K   K   M
gct tca gca agt act ggc gag atg gag ccg ttg agt ccg gaa cga cca ctc agt ccg acc
 A   S   A   S   T   G   E   M   E   P   L   S   P   E   R   P   L   S   P   T
aat gcg gga agt ggg gct gcc tca cct ttt aac caa gct aag gaa caa gtt agc ccc ggt
 N   A   G   S   G   A   A   S   P   F   N   Q   A   K   E   Q   V   S   P   G
gct gta cct cca aca ggt ctt gag aag ctt gtc aac aag ctc atg gac gct tca gag gct
 A   V   P   P   T   G   L   E   K   L   V   N   K   L   M   D   A   S   E   A
acc ctc tca agt caa cca gct gag gcc gtg acg ccc gaa cag aaa ctg gct gaa aaa ctt
 T   L   S   S   Q   P   A   E   A   V   T   P   E   Q   K   L   A   E   K   L
gaa aaa ttg gga ctg gca cct gcg tct acc act agt gcg ccg cca ccg cac cca aaa gtt
 E   K   L   G   L   A   P   A   S   T   T   S   A   P   P   P   H   P   K   V
gct gct ctg aat gcg cag tct gtc gaa gat gcg cgg aag act tct acc cat gcc ctt tac
 A   A   L   N   A   Q   S   V   E   D   A   R   K   T   S   T   H   A   L   Y
tcc gtt gat taa
 S   V   D   -
```

Supplementary Fig. 6. Codon-optimized sequence of *Capsaspora* NF-κB with the corresponding amino acids.

```
atg gaa gcc aga tgg cgg cgg gcc cct ggc ctg ggc aac cct cgg cct agc aag cgg tgc
 M   E   A   R   W   R   R   A   P   G   L   G   N   P   R   P   S   K   R   C
aga agc cag agc cct agc caa aga cag gtg atc gcc gag tct ttc ggc aag ggc cag gcc
 R   S   Q   S   P   S   Q   R   Q   V   I   A   E   S   F   G   K   G   Q   A
gct gcc gaa gag gac gcc aga ggc gag gcc ggc ccc gcc gcc atg gtc cct ctg gga ggc
 A   A   E   E   D   A   R   G   E   A   G   P   A   A   M   V   P   L   G   G
gcc atc gac ttc acc gac gtt ggc atg gcc gct tct agc ttt gac ccc gcc gtg tgg cac
 A   I   D   F   T   D   V   G   M   A   A   S   S   F   D   P   A   V   W   H
ggc atg gtg gcc aag agc acc atg ctg gcc caa cag ggc cac aga gga att tct ctg aac
 G   M   V   A   K   S   T   M   L   A   Q   Q   G   H   R   G   I   S   L   N
gac atc gca gtg ggc gga ttc gac gac acc gtg tcc gag gtg gac ggc gtg ctg gaa atc
 D   I   A   V   G   G   F   D   D   T   V   S   E   V   D   G   V   L   E   I
agc cag cag cct gcc gcc cac gga aga ttc aga tac agc aag gag ggc aga aag aca cct
 S   Q   Q   P   A   A   H   G   R   F   R   Y   S   K   E   G   R   K   T   P
ctg cct gga gct atc gag ggc agc ttc ccc acc gtg cag ctg agc ccc gct tat aga cac
 L   P   G   A   I   E   G   S   F   P   T   V   Q   L   S   P   A   Y   R   H
atg gtg ccc gac ggc acc atg gtt tct gtg acc ctg atc acc aag tac cac gat gat aga
 M   V   P   D   G   T   M   V   S   V   T   L   I   T   K   Y   H   D   D   R
ggc gct ccg gtt aga cac tgg cac acc ctc gag gtg aag gaa ggc ggc gcc gcc tct aga
 G   A   P   V   R   H   W   H   T   L   E   V   K   E   G   G   A   A   S   R
cct ctg agc atg ggc ctg gtg cag ttc cct aat ctg gtc gtg acc aga gcc gct atg cct
 P   L   S   M   G   L   V   Q   F   P   N   L   V   V   T   R   A   A   M   P
gct gat ctg gac gat gct gga gct gca aga aac ccg gaa gat cag cac gtg att aga ctg
 A   D   L   D   D   A   G   A   A   R   N   P   E   D   Q   H   V   I   R   L
atg ttt aca atg atg ttc cgg gac agc gcc gga gtg atc ttc aag acc agc gtg atc agc
 M   F   T   M   M   F   R   D   S   A   G   V   I   F   K   T   S   V   I   S
aat cct atc tac ggc atc gag ctg aag atc cac aag tcc agc cac ccc aga gtg cct gtg
 N   P   I   Y   G   I   E   L   K   I   H   K   S   S   H   P   R   V   P   V
gcc ggc cag ctc gac gtc ttt ttc ctg aca tct aag gtg aaa aag aaa aac acc ctc atc
 A   G   Q   L   D   V   F   F   L   T   S   K   V   K   K   K   N   T   L   I
aag gtg aga gag gtg tac ccc gct cct ttc gat cct ggc cca aac agc ggc tgg gag ctg
 K   V   R   E   V   Y   P   A   P   F   D   P   G   P   N   S   G   W   E   L
gac gag aga ggc aga ctg acc ttc acc gtg gac aac ctg cac gtg cac tac cag tac gcc
 D   E   R   G   R   L   T   F   T   V   D   N   L   H   V   H   Y   Q   Y   A
gtg gtc gcc aca gtg cca cct tac tgg gac cag aca atc aca aca agc aga gtg ctg gac
 V   V   A   T   V   P   P   Y   W   D   Q   T   I   T   T   S   R   V   L   D
atc tgc ctg gtg gat acc gcc cag gga ctg gaa agc aac tgc gtg cag atc gag tac tgc
 I   C   L   V   D   T   A   Q   G   L   E   S   N   C   V   Q   I   E   Y   C
ccc ccc agc gcc gca gaa ggc gaa ggc gct gct gcc gcc gct tct ggc gcc gct agt gat
 P   P   S   A   A   E   G   E   G   A   A   A   A   A   S   G   A   A   S   D
gcc gcc cct ggc cct gcc ctg atg tga
 A   A   P   G   P   A   L   M   -
```

Supplementary Fig. 7. Codon-optimized sequence of *As*-NF-κB1 with the corresponding amino acids.

```
atg gag ccc tgg cga aca cgc caa tac tca caa aaa cgg tca aga act gag agc ccg ccc
 M   E   P   W   R   T   R   Q   Y   S   Q   K   R   S   R   T   E   S   P   P
acc ggc atc gcc atg tct cca cat aaa gtg ctc ggc ttc ggc ggg gca gcc gca gcc gca
 T   G   I   A   M   S   P   H   K   V   L   G   F   G   G   A   A   A   A   A
gca gaa atg gaa gaa ggt ggc gct gcc gta acc ggc cat gtg cct ctg ggg ccg ttc gga
 A   E   M   E   E   G   G   A   A   V   T   G   H   V   P   L   G   P   F   G
ggt atg caa tcc ctg aca atg cac cac ttc gac ttg ccg gac ttg tct gat cct agg acg
 G   M   Q   S   L   T   M   H   H   F   D   L   P   D   L   S   D   P   R   T
cct ctg cgg ctt gat gac ctc acg gaa aaa ccg cct cca ccg ccg cac gat gcc att cct
 P   L   R   L   D   D   L   T   E   K   P   P   P   P   P   H   D   A   I   P
ccc tgg cct aat aca ttc gga gct ggg aaa tcc cct gtt ggc ccc ttg cca ccc cct cat
 P   W   P   N   T   F   G   A   G   K   S   P   V   G   P   L   P   P   P   H
aaa cca aca aag gca gag ccc atg tct atc agt gaa ccg agt tca cct cgc aat gac gca
 K   P   T   K   A   E   P   M   S   I   S   E   P   S   S   P   R   N   D   A
agc aga gtc gtg gag atg tca gct gaa ggc tat cca ata gta tct ggg gtt ctt aga atc
 S   R   V   V   E   M   S   A   E   G   Y   P   I   V   S   G   V   L   R   I
gtc cag caa cct gcg tct cac ggg cgg ttt aga tat tca aaa gaa ggt agg aag acg ccg
 V   Q   Q   P   A   S   H   G   R   F   R   Y   S   K   E   G   R   K   T   P
ctc cat gga cgg gag gac ggc agc tat ccc acg gta gca atc gcc gat agg tac agg cac
 L   H   G   R   E   D   G   S   Y   P   T   V   A   I   A   D   R   Y   R   H
ctg gtg gag gaa ggt acg caa gta gat gtc acc ctc gta acc aaa cat aac gac gaa cat
 L   V   E   E   G   T   Q   V   D   V   T   L   V   T   K   H   N   D   E   H
gga tct ccc att caa cac tgg cat gtt ctt gaa gga aaa gaa ggt ggc cca gtc tct cgg
 G   S   P   I   Q   H   W   H   V   L   E   G   K   E   G   G   P   V   S   R
cct ctg aag gac ggt gtc gcg aca ttc cca aat ctg gtg gta act cgc acc act gcg gaa
 P   L   K   D   G   V   A   T   F   P   N   L   V   V   T   R   T   T   A   E
aaa gag gga ggt gcg cga aac gta gag gat cag cat gtt atc agg ctg atg tat aca atg
 K   E   G   G   A   R   N   V   E   D   Q   H   V   I   R   L   M   Y   T   M
cgg ttt cag gac gag aaa agg aga agc gtg ctg gcg agg gcg ata agt gaa ccc atc ttt
 R   F   Q   D   E   K   R   R   S   V   L   A   R   A   I   S   E   P   I   F
ggt caa gag gtc aag atc cac cgc att tct cac atg cag att cct gcc acg gga aat atc
 G   Q   E   V   K   I   H   R   I   S   H   M   Q   I   P   A   T   G   N   I
gaa gtc ttt ttc ttg acc tcc aaa att aag cgg aag aat act ata ttg act ttc act gag
 E   V   F   F   L   T   S   K   I   K   R   K   N   T   I   L   T   F   T   E
acc aca cca tca agt ttt gat ccg ggg cca cga tcc aag tgg cag ttg gac aaa aat atg
 T   T   P   S   S   F   D   P   G   P   R   S   K   W   Q   L   D   K   N   M
tgt ttg aca tac gcc atg aag gat ttg acg gtt cat tat caa tat gct gta gtg gct cga
 C   L   T   Y   A   M   K   D   L   T   V   H   Y   Q   Y   A   V   V   A   R
gtc cct ccc tat tgg gac cag acg atc acc tct ccg cgg agg att aaa gtg tgc ctc ata
 V   P   P   Y   W   D   Q   T   I   T   S   P   R   R   I   K   V   C   L   I
gac acc gtc caa ggc ctg gaa tca aat gta gtt gaa att gat tat gtc cct gct ccg ttc
 D   T   V   Q   G   L   E   S   N   V   V   E   I   D   Y   V   P   A   P   F
cga cca tac tct tcc gcg tct gct cct cct ggc cac cca cca gtc ttc ggc atg gca cct
 R   P   Y   S   S   A   S   A   P   P   G   H   P   P   V   F   G   M   A   P
tct gtc cat gga tct tgg gga gtt cct caa ctc gat tgg ttc ccg gcg gat aat gct gct
 S   V   H   G   S   W   G   V   P   Q   L   D   W   F   P   A   D   N   A   A
ggg gcc gct gca gat atg ccc atg agg gat tcc agt aat tct cca tct gta agc gcg tca
 G   A   A   A   D   M   P   M   R   D   S   S   N   S   P   S   V   S   A   S
tcc gaa tcc aca gat aca cga cct tct cgc ggg agg gta tac ggg tcc ttc gag act tcc
 S   E   S   T   D   T   R   P   S   R   G   R   V   Y   G   S   F   E   T   S
gtt taa
 V   -
```

Supplementary Fig. 8. Codon-optimized sequence of *As*-NF-κB2 with the corresponding amino acids.

atg cca aat gat ttc gag gct gtt ctg acg tct ggg ggt ggg tct cat gct tgc gga cca
M P N D F E A V L T S G G G S H A C G P
tct gcc gcc aag aga gct agg gtg gac ttg agt gat gcc tcc gaa aac gcc tat aac cag
S A A K R A R V D L S D A S E N A Y N Q
gca tgg aga ccc tcc gca tct gtg gct tgt gga gct acc ttt cag cca cca agg aca ccc
A W R P S A S V A C G A T F Q P P R T P
atg act gca tgg gac ctg ggc att ctt cct cct aca act cat aca ggc cac gga gga ttc
M T A W D L G I L P P T T H T G H G G F
cca ccg cga gca tac ccc att gag tct ttc cct atg cat cga ggt ttc ctt cag gtt agt
P P R A Y P I E S F P M H R G F L Q V S
gga gct gca gca gcc ccg cca ata ccg aga ttg atg ggg ggc cac cat cag tac cac cct
G A A A A P P I P R L M G G H H Q Y H P
aca cac gcc atg tat cgc gat ctg cgg gaa gtc ttg gaa att acc gag cag ccg act gaa
T H A M Y R D L R E V L E I T E Q P T E
aga ggg cgg ttc aga tac gca aag gaa aaa aga cga acc cca ctc ccg ggg aga agg gat
R G R F R Y A K E K R R T P L P G R R D
gga gga ttt ccc acg gtt agg gtt gct gac aga tat cga gac gtc ttg cca gat ggc act
G G F P T V R V A D R Y R D V L P D G T
cac ata cag gca tcc gtg gtg acg aga cag gac gga gaa gac ggg atc cca cgc cct cac
H I Q A S V V T R Q D G E D G I P R P H
tgg cac aga ctc gag gga agg gaa gga gag agc gtt agc cag cct ctc agc ctg gga gcc
W H R L E G R E G E S V S Q P L S L G A
gct aca ttc tcc aac ctc gtg gtg atc cgg agc gac aga aat gcc gca gaa aat cac tgg
A T F S N L V V I R S D R N A A E N H W
ggc ccc agg cct acc gaa gac caa cag gtg atc cga att atg ttt tcc gtg ctt ttc cgg
G P R P T E D Q Q V I R I M F S V L F R
ttg cct acc ggt gaa atg gcc cgg tct tgg gtg gct tcc gaa cct atc tac ggg tgc gac
L P T G E M A R S W V A S E P I Y G C D
ctg aaa att cag aac atg tct cac aca gaa gtc ccg atg ctc tcc ggt gct gag gtg acc
L K I Q N M S H T E V P M L S G A E V T
ctg ctg act agt aag atc agg aaa cag tcc atc gcc ctg atg ttg gtt gat gaa ttt aca
L L T S K I R K Q S I A L M L V D E F T
gat cat gac ttc gct ccc gga aca aag att gac cca gca aat ggg tgg gag tgt gat cag
D H D F A P G T K I D P A N G W E C D Q
gac ggg aaa atc tgt tgc act att cag ccc agc tac gtg cac cat cag tac gct ctc gtg
D G K I C C T I Q P S Y V H H Q Y A L V
gcg cag ata cca cag tat tgg gac ctg acc ttg gag tct gac aga caa att agc gcc agg
A Q I P Q Y W D L T L E S D R Q I S A R
att atc gat aag gat cag aag atg gag tcc aac tgt tct caa ctc aca tat gtc ccc gga
I I D K D Q K M E S N C S Q L T Y V P G
tct gag gcc aga agg cgc aga cca cca ccc gaa tga
S E A R R R R P P P E -

Supplementary Fig. 9. Codon-optimized sequence of *As*-NF-κB3 with the corresponding amino acids.

Fig. 2b

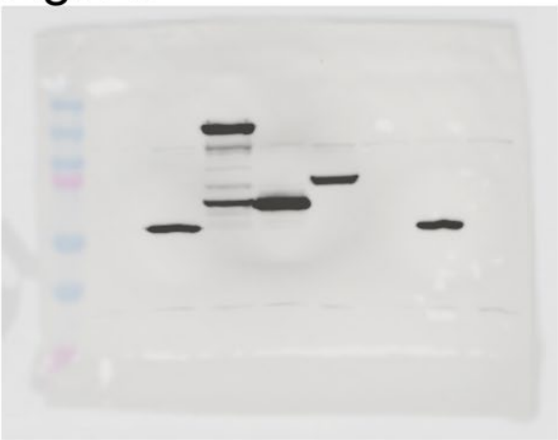

Fig. 2d (reverse image)

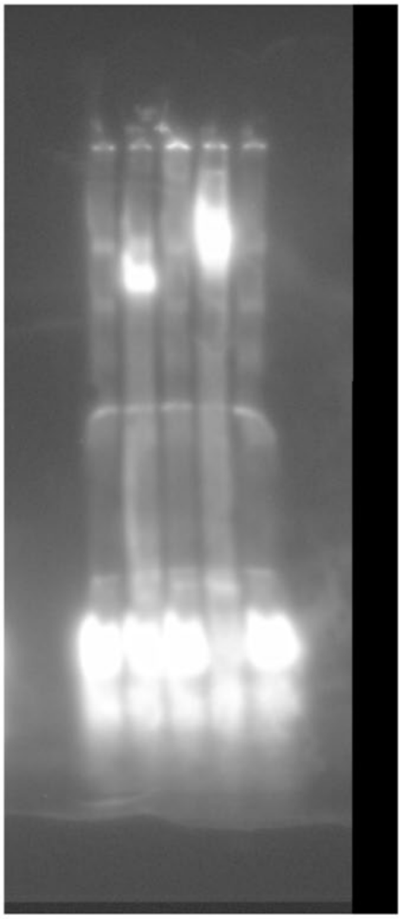

Fig. 2g

Anti-MYC

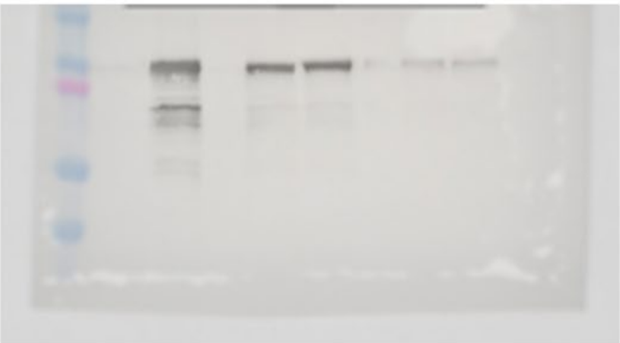

Anti-FLAG

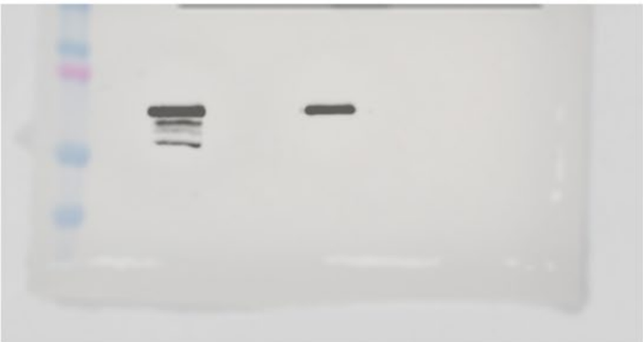

Fig. 3a

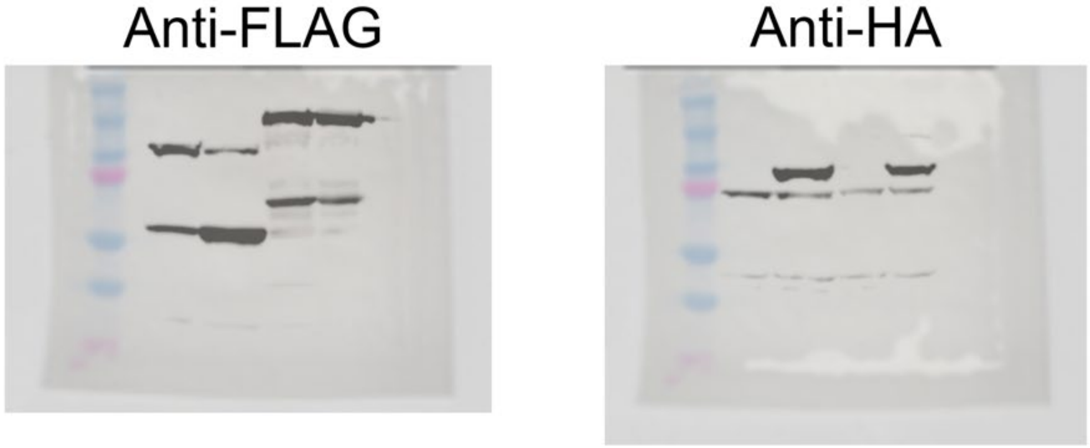

Fig. 3b

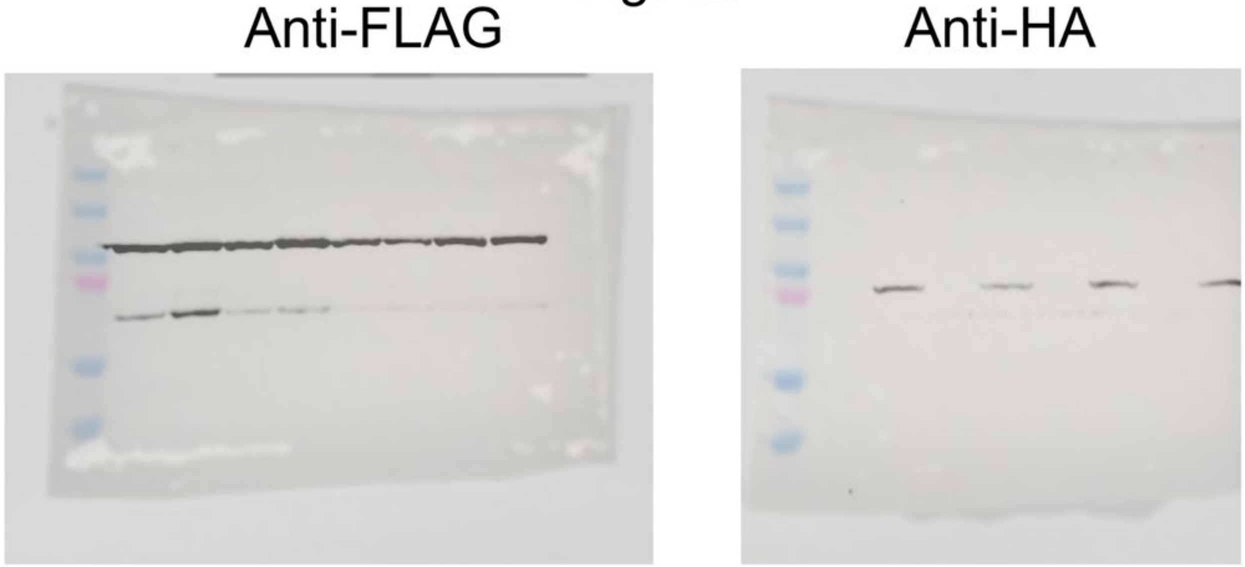

Supplementary Fig. 11. Uncropped versions of Figures 3a and 3b.

Fig. 5d

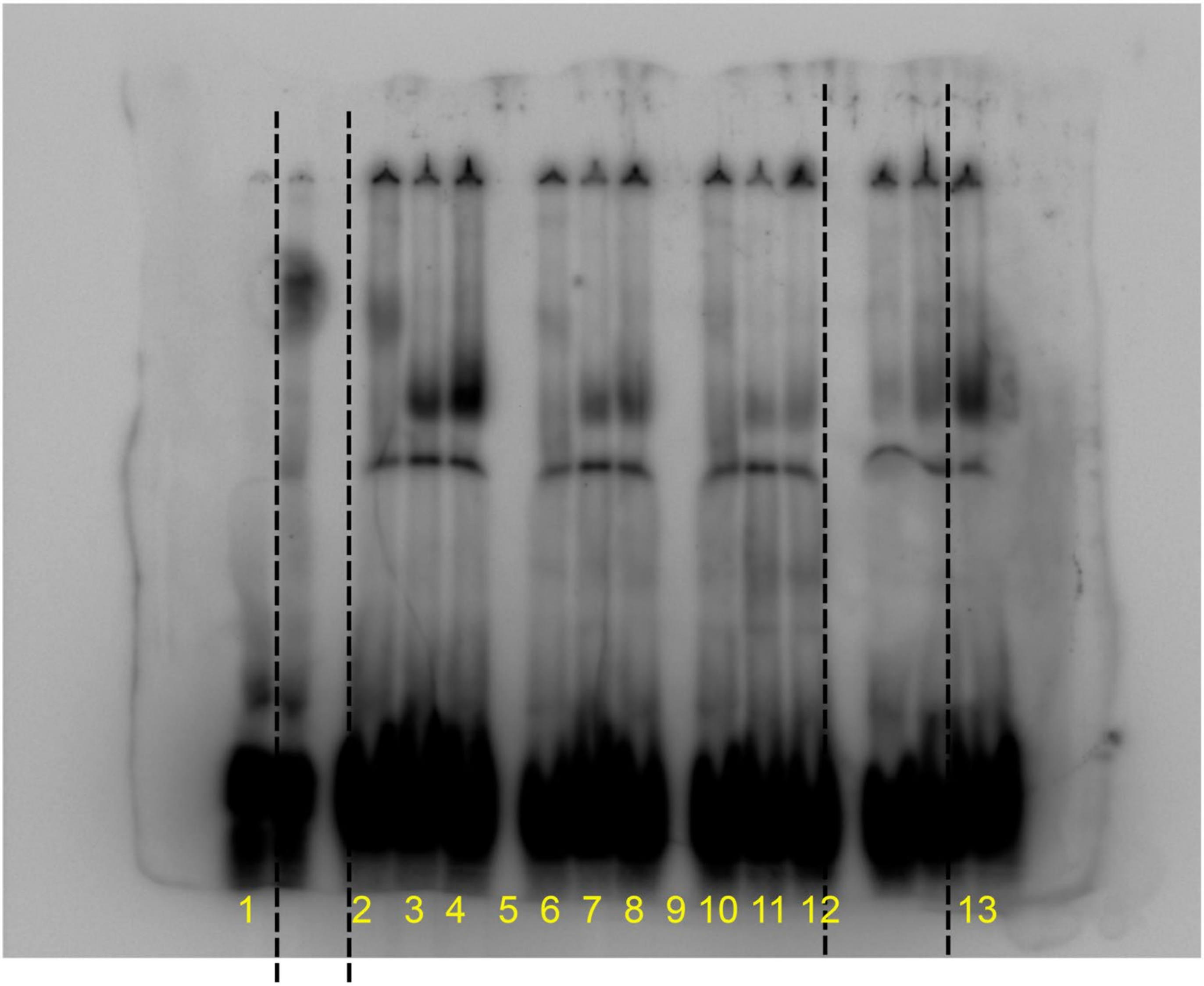

Supplementary Fig.12. Uncropped version of Fig. 5d. Dotted lines indicate where the image was cropped, and numbers correspond to the lanes used in Fig. 5d.

Fig. 6b

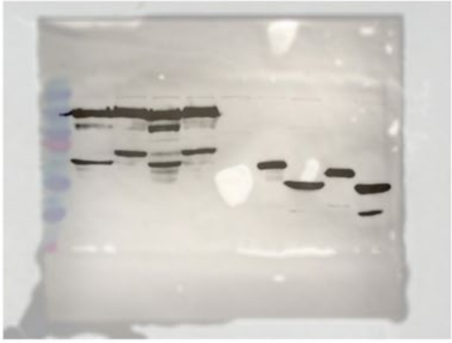

Fig. 6g

Anti-MYC Anti-FLAG

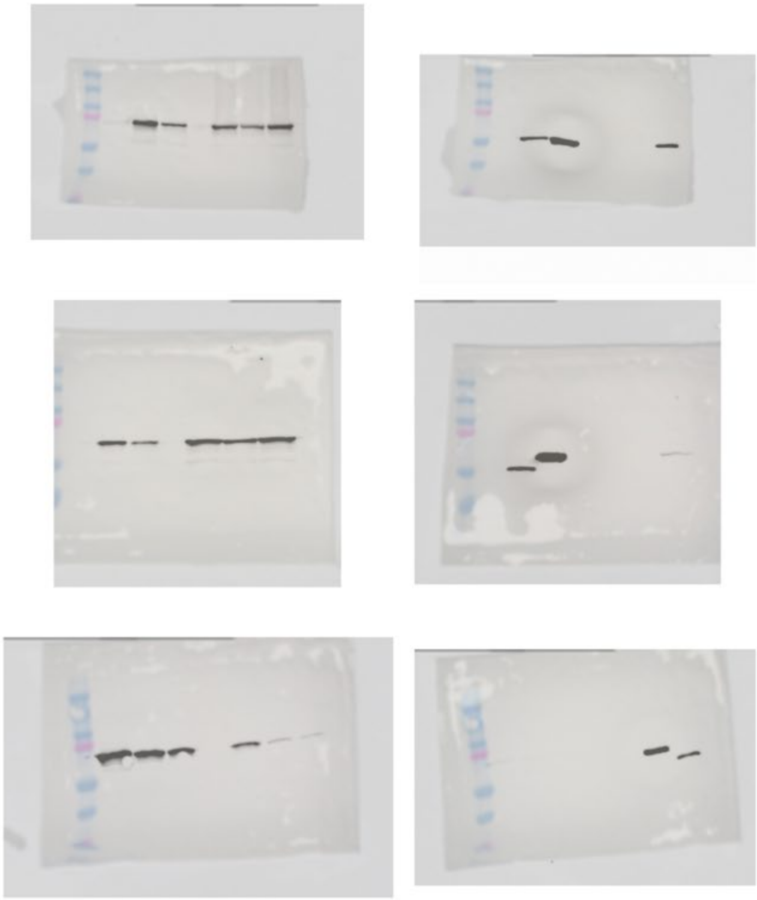

Fig. 6d (reverse image)

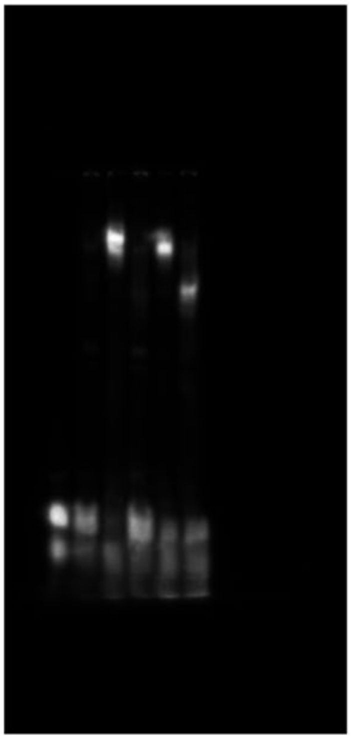

Supplemental Fig. 3a

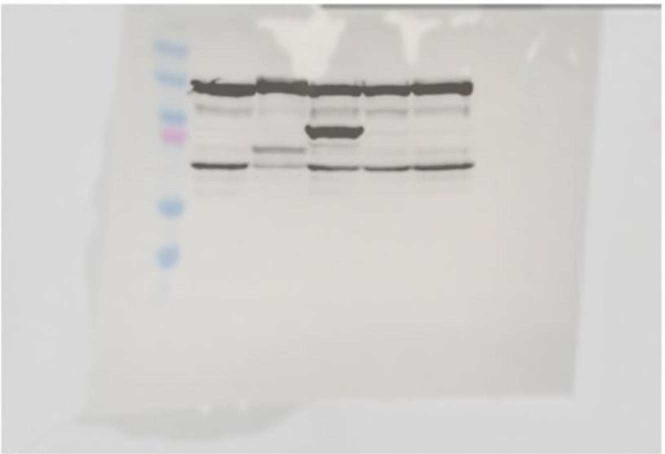

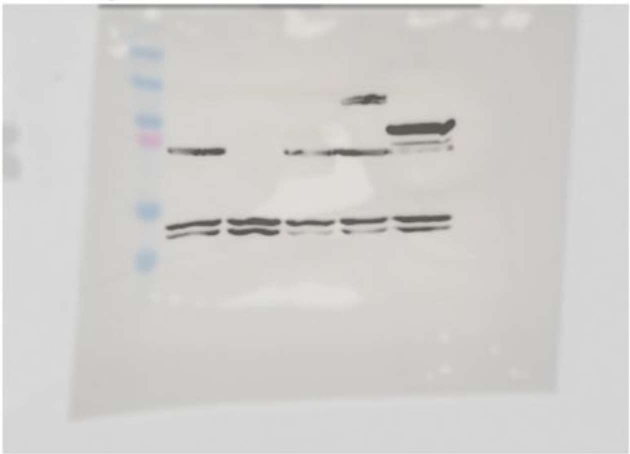

Supplemental Fig. 3b

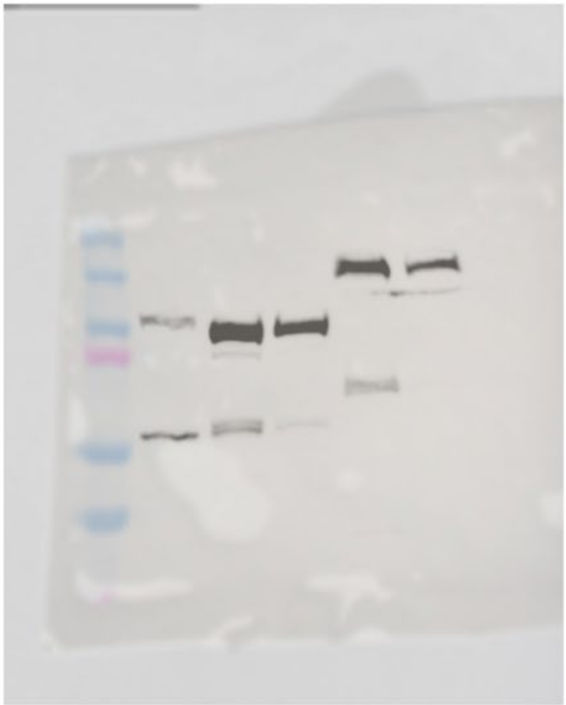

Supplementary References

1. Wolenski, F.S. *et al*. Characterization of the core elements of the NF-κB signaling pathway of the sea anemone *Nematostella vectensis*. *Mol. Cell. Biol.* **31**, 1076-1087 (2011).

2. Mansfield, K.M. *et al*. Transcription factor NF-κB is modulated by symbiotic status in a sea anemone model of cnidarian bleaching. *Sci. Rep.* **7**, 16025 (2017).

3. Richter, D.J., Fozouni, P., Eisen, M. B. & King, N. Gene family innovation, conservation and loss on the animal stem lineage. *eLife* **7**, e34226 (2018).

4. Williams, L.M. & Gilmore, T.D. Looking down on NF-κB. *Mol. Cell. Biol.* **40**, e00104-20 (2020).
