# Supplementary material for: Comparison of NF-κB from the protists Capsaspora owczarzaki and Acanthoeca spectabilis reveals extensive evolutionary diversification of this transcription factor
Source: Commun Biol. 2021 Dec 16;4:1404. doi: 10.1038/s42003-021-02924-2 (PMC8677719; doi:10.1038/s42003-021-02924-2)
Supplement: Supplementary file 2 — Description of Additional Supplementary Files [file 42003_2021_2924_MOESM2_ESM.pdf]

Description of Additional Supplementary Files

File name: Supplementary Data 1.
Description: Protein Binding Microarray data from ref (24). Each value is a Z-score for the indicated probe and protein. The sequences most similar to the kappaB site EMSA probe are listed on the right.

File name: Supplementary Data 2.
Description: Sheet 1 is all 1348 genes that are expressed in the same way as NF-κB across the three life stages of Capsaspora. Sheet 2 lists the 389 annotated genes from the 1348 genes shown in sheet 1, and their corresponding UniProt IDs.

File name: Supplementary Data 3.
Description: PBM-generated Co-NF-κB motif search results (Sheet 2) from MEME-FIMO and total Co-NF-κB motif sites (Sheet 3). Also a list of the 500 bp Page 7 of 21 upstream sequences of the 1348 genes that express in the same pattern as NF-kB over the three life stages (Sheet 4).

File name: Supplementary Data 4.
Description: RHD sequences of organisms used for Maximum Likelihood phylogenetic analysis.

File name: Supplementary Data 5.
Description: Supplementary Data 5. Raw data for information in Figs. 2e, 2f, 5c, 6e, and 6f.
