# Supplementary material for: Comparison of NF-κB from the protists Capsaspora owczarzaki and Acanthoeca spectabilis reveals extensive evolutionary diversification of this transcription factor
Source: Commun Biol. 2021 Dec 16;4:1404. doi: 10.1038/s42003-021-02924-2 (PMC8677719; doi:10.1038/s42003-021-02924-2)
Supplement: Supplementary file 8 — Reporting Summary [file 42003_2021_2924_MOESM8_ESM.pdf]

nature portfolio

Corresponding author(s): Thomas Gilmore

Last updated by author(s): Oct 14, 2021

Reporting Summary

Nature Portfolio wishes to improve the reproducibility of the work that we publish. This form provides structure for consistency and transparency in reporting. For further information on Nature Portfolio policies, see our Editorial Policies and the Editorial Policy Checklist.

Statistics

For all statistical analyses, confirm that the following items are present in the figure legend, table legend, main text, or Methods section.

n/a	Confirmed	
☐	☒	The exact sample size (n) for each experimental group/condition, given as a discrete number and unit of measurement
☐	☒	A statement on whether measurements were taken from distinct samples or whether the same sample was measured repeatedly
☒	☐	The statistical test(s) used AND whether they are one- or two-sided<br>*Only common tests should be described solely by name; describe more complex techniques in the Methods section.*
☒	☐	A description of all covariates tested
☒	☐	A description of any assumptions or corrections, such as tests of normality and adjustment for multiple comparisons
☐	☒	A full description of the statistical parameters including central tendency (e.g. means) or other basic estimates (e.g. regression coefficient) AND variation (e.g. standard deviation) or associated estimates of uncertainty (e.g. confidence intervals)
☒	☐	For null hypothesis testing, the test statistic (e.g. F, t, r) with confidence intervals, effect sizes, degrees of freedom and P value noted<br>*Give P values as exact values whenever suitable.*
☒	☐	For Bayesian analysis, information on the choice of priors and Markov chain Monte Carlo settings
☒	☐	For hierarchical and complex designs, identification of the appropriate level for tests and full reporting of outcomes
☒	☐	Estimates of effect sizes (e.g. Cohen's d, Pearson's r), indicating how they were calculated

Our web collection on statistics for biologists contains articles on many of the points above.

Software and code

Policy information about availability of computer code

Data collection	No software was used.
Data analysis	No software was used.

For manuscripts utilizing custom algorithms or software that are central to the research but not yet described in published literature, software must be made available to editors and reviewers. We strongly encourage code deposition in a community repository (e.g. GitHub). See the Nature Portfolio guidelines for submitting code & software for further information.

Data

Policy information about availability of data

All manuscripts must include a data availability statement. This statement should provide the following information, where applicable:

- Accession codes, unique identifiers, or web links for publicly available datasets
- A description of any restrictions on data availability
- For clinical datasets or third party data, please ensure that the statement adheres to our policy

All data generated or analyzed during this study are included in this published article (and its supplementary information files).

Field-specific reporting

Please select the one below that is the best fit for your research. If you are not sure, read the appropriate sections before making your selection.

☒ Life sciences ☐ Behavioural & social sciences ☐ Ecological, evolutionary & environmental sciences

For a reference copy of the document with all sections, see nature.com/documents/nr-reporting-summary-flat.pdf

Life sciences study design

All studies must disclose on these points even when the disclosure is negative.

Sample size	Samples sizes for experiments such as immunofluorescence and reporter gene assays were chosen to give statistically significant data according to standard experiments in the field. For example, with immunofluorescence, we analyzed over 100 cells per sample from multiple fields, and for reported gene assays, experiments were from at least three biologically independent experiments performed with triplicate samples.
Data exclusions	In general, no data were excluded. However, for analysis of RNA-Seq data for transcripts that covaried with NF-kB expression, we excluded RNAs that had RFKM values of zero, as reported in the Methods.
Replication	Experiments were replicated and appropriate measures were taken to verify reproducibility.
Randomization	This is not relevant to our study because we used cell lines in culture, and did not use animals, humans, or otherwise independent samples.
Blinding	Blinding is not relevant to our study, as all experiments involved direct comparision of data between samples in the given group, e.g., for control vectors in Western blots, EMSAs, reporter gene assays, immunoprecipitations, and indirect immunofluorescence.

Reporting for specific materials, systems and methods

We require information from authors about some types of materials, experimental systems and methods used in many studies. Here, indicate whether each material, system or method listed is relevant to your study. If you are not sure if a list item applies to your research, read the appropriate section before selecting a response.

Materials & experimental systems

n/a	Involved in the study	
☐	☒	Antibodies
☐	☒	Eukaryotic cell lines
☒	☐	Palaeontology and archaeology
☒	☐	Animals and other organisms
☒	☐	Human research participants
☒	☐	Clinical data
☒	☐	Dual use research of concern

Methods

n/a	Involved in the study	
☒	☐	ChIP-seq
☒	☐	Flow cytometry
☒	☐	MRI-based neuroimaging

Antibodies

Antibodies used	anti-FLAG antiserum (Cell Signaling Technology, #2368), anti-HA antiserum (Santa Cruz Biotechnology, #sc-805), anti-MYC antiserum (Cell Signaling Technology, #71D10)
Validation	Antibodies were verified by using negative controls in experiments (e.g., vector alone) and the identification of specific bands in Western blots of the predicted sizes. The anti-FLAG antibody is a rabbit monoclonal antibody that binds to the same epitope as the Anti-FLAG M2 antibody, and has approved applications in Western blotting, Immunoprecipitation, and Immunofluorescence. The anti-MYC antibody is a rabbit monoclonal antibody that has approved applications for Western blotting, Immunoprecipitation, and Immunofluorescence. The anti-HA is a rabbit polyclonal antibody, and has approved applications for Western Blotting, Immunoprecipitation, and Immunofluorescence.

Eukaryotic cell lines

Policy information about cell lines

Cell line source(s)	HEK 293T (ATCC); HEK 293 (ATCC), DF-1 chicken fibroblasts (David Foster, Univ of Minnesota, doi: 10.1006/viro.1998.9290); Capsaspora (ATCC)
Authentication	Cell lines were authenticated by suppliers.

Mycoplasma contamination	Cell lines were not tested for mycoplasma contamination. However, the experiments performed herein did not characterize specific properties of the cells, rather they used the cells as vehicles for overexpressing exogenous proteins.
Commonly misidentified lines<br>(See ICLAC register)	None.
